# Vascular Patterns in Iguanas and Other Squamates: Blood Vessels and Sites of Thermal Exchange

**DOI:** 10.1371/journal.pone.0139215

**Published:** 2015-10-14

**Authors:** William Ruger Porter, Lawrence M. Witmer

**Affiliations:** Department of Biomedical Sciences, Heritage College of Osteopathic Medicine, Ohio University, Athens, Ohio, United States of America; Tokyo Medical and Dental University, JAPAN

## Abstract

Squamates use the circulatory system to regulate body and head temperatures during both heating and cooling. The flexibility of this system, which possibly exceeds that of endotherms, offers a number of physiological mechanisms to gain or retain heat (e.g., increase peripheral blood flow and heart rate, cooling the head to prolong basking time for the body) as well as to shed heat (modulate peripheral blood flow, expose sites of thermal exchange). Squamates also have the ability to establish and maintain the same head-to-body temperature differential that birds, crocodilians, and mammals demonstrate, but without a discrete rete or other vascular physiological device. Squamates offer important anatomical and phylogenetic evidence for the inference of the blood vessels of dinosaurs and other extinct archosaurs in that they shed light on the basal diapsid condition. Given this basal positioning, squamates likewise inform and constrain the range of physiological thermoregulatory mechanisms that may have been found in Dinosauria. Unfortunately, the literature on squamate vascular anatomy is limited. Cephalic vascular anatomy of green iguanas (*Iguana iguana*) was investigated using a differential-contrast, dual-vascular injection (DCDVI) technique and high-resolution X-ray microcomputed tomography (μCT). Blood vessels were digitally segmented to create a surface representation of vascular pathways. Known sites of thermal exchange, consisting of the oral, nasal, and orbital regions, were given special attention due to their role in brain and cephalic thermoregulation. Blood vessels to and from sites of thermal exchange were investigated to detect conserved vascular patterns and to assess their ability to deliver cooled blood to the dural venous sinuses. Arteries within sites of thermal exchange were found to deliver blood directly and through collateral pathways. The venous drainage was found to have multiple pathways that could influence neurosensory tissue temperature, as well as pathways that would bypass neurosensory tissues. The orbital region houses a large venous sinus that receives cooled blood from the nasal region. Blood vessels from the nasal region and orbital sinus show anastomotic connections to the dural sinus system, allowing for the direct modulation of brain temperatures. The generality of the vascular patterns discovered in iguanas were assessed by firsthand comparison with other squamates taxa (e.g., via dissection and osteological study) as well as the literature. Similar to extant archosaurs, iguanas and other squamates have highly vascularized sites of thermal exchange that likely support physiological thermoregulation that “fine tunes” temperatures attained through behavioral thermoregulation.

## Introduction

The cranial vascular anatomy of squamates has received much attention in the past, often in a very broad taxonomic context. Bruner [1] investigated the veins in the lizard *Lacerta agilis*, the snake *Tropidonotus natrix*, and the turtle *Emys orbicularis*. O’Donoghue [2] and Dendy [3] looked at nearly the entire vascular system in the tuatara *Sphenodon punctatus*, and Heath [4, 5] investigated blood vessels in *Phrynosoma coronatum*. Burda [6] studied the developmental changes of blood vessels in *Crotaphytus collaris* and *Podarcis muralis*. These works, along with many others, have formed a solid foundation for studies of the role of blood vessels in thermal physiology, but studying the physiological and other functional roles of blood vessels in reptiles can be a complex matter [7]. For example, *Phrynosoma* uses blood vessels in defense [8] by squirting blood from the orbital region. Additionally, lizards use the orbital sinuses during ecdysis [1] and to aid in clearing debris from the orbital margins [5]. Another example of this complexity in lizards is that, being ectotherms, the circulatory system may be more optimized for regulating the transfer of thermal energy rather than for oxygen delivery [9]. Reptiles have a number of vascular capabilities to control temperature such as increasing peripheral blood flow and, consequently, heart rate, during basking to increase delivery of warmed blood to the core [10–16]. Spray and Belkin [16] noted that the heart warmed up faster than the body in iguanids, implying warmed peripheral blood flows through veins to the heart relatively quickly. Other studies have shown additional physiological abilities, including decreases in peripheral blood flow and heart rate during cooling that slows heat loss to the environment [10, 15], a low hematocrit that offers lower resistance to flow and is less energetically costly to pump [9], and a muscle in the internal jugular vein [1] that restricts venous flow to the body from head [4, 5, 17]. To make physiological thermoregulation even more complex, differing inter- and intraspecific thermoregulatory abilities and preferences are found among lizards, and variations in individual behavior can influence thermoregulatory patterns [18].

The bulk of the research in squamate thermoregulation has focused on behavioral thermoregulation, which has a rich history that has clearly demonstrated that squamates indeed use specific behaviors to draw on extrinsic environmental parameters to modulate their internal thermal environment [19, 20]. Fewer studies focused on the intrinsic physiological thermoregulatory capabilities in reptiles that allow squamates to “fine tune” or subtly influence temperature control. The earlier works on physiological thermoregulation in squamates focused on the ability to establish a head-to-body temperature differential [1, 4, 10, 18] indicating that head temperature can be controlled within narrow limits [18, 21–23]. Typically, when squamates first exit their refugium, head and body temperatures are roughly equivalent [4, 5, 24, 25]. Usually, only the head is exposed [4, 22], allowing the head to warm up while still remaining partially covered. In *Dipsosaurus* and *Phrynosoma*, when the head was around 2°C warmer than the body, the eyes bulged [4, 5, 24, 25]. Activation of the jugular constrictor muscle has been shown to increase venous pressure which causes the eyes to bulge. According to Heath’s [4] hypothesis, counter-current heat exchange occurs between the internal jugular vein and internal carotid artery, and when the jugular constrictor muscle is not activated, the counter-current heat exchanger establishes a temperature gradient along the internal carotid artery’s length, allowing the head to warm faster than the body. When the jugular constrictor muscle contracts, venous flow through the internal jugular vein decreases and the counter-current heat exchange stops, and any blood warmed in the head is shunted directly to the body through the *external* jugular vein [4]. Then, cooler, body-temperature blood reaches the head, slowing the warming of the head and increasing the warming of the body, resulting in equilibration of head and body temperatures. After a few minutes after contraction of the jugular constrictor muscle, the eye bulging is reduced and the head and body are the same temperature [4, 24]. Crawford [26] offered another hypothesis as to the role of the jugular constrictor muscle. In *Sauromalus*, the eyes bulge during panting, implying a more general role in thermoregulation. Crawford [26] hypothesized that if blood were cooled in the internal carotid artery during panting, then cooled carotid blood would be warmed by the draining of warmed venous blood in the internal jugular vein, reducing the efficiency of panting and its impact on brain cooling. Crawford [26] offered an alternative hypothesis: when the jugular constrictor muscle contracts, the cool carotid blood from the body would gain heat by flowing over the brain, instead of gaining heat from the internal jugular vein, to facilitate brain cooling. Crawford [26] reported that activation of the jugular constrictor muscle during heating can diminish the head-to-body temperature differential, but during panting can reverse the gradient and facilitate brain cooling, indicating a complex role of the jugular constrictor muscle during heating and cooling, especially during evaporative cooling.

Physiological thermoregulation involving evaporative cooling has been repeatedly demonstrated in squamates [1, 17]. Anole, basilisk, and *Podarcis* lizards can maintain head-to-body temperature gradients using evaporative cooling [17, 26, 27]. *Sceloporus* can keep its brain cooler than its body during both rest and exercise [28], and Spray and Belkin [16] showed that airway passage temperatures during cooling were lower than that of the surrounding air. This evidence implies that evaporative cooling, in conjunction with circulatory modulation, allows for physiological fine tuning of temperatures within the head.

Squamates routinely use the oral, nasal, and orbital regions as sites of thermal exchange (a trait shared with archosaurs [22, 29]). For example, Borrell et al. [30] offered evidence that rattlesnakes use evaporative cooling within the nasal region, and this head-to-body temperature differential was increased at lower humidity, giving additional evidence for evaporative cooling supporting temperature differentials. Additionally, Cadena et al. [31] showed evidence in crotalid snakes that evaporative cooling within the nasal passages ultimately influences the temperature of the entire rostral portion of the head. The temperature of air exhaled through the nasal region in *Dipsosaurus* was found to be lower than body temperature, indicating evaporative cooling and water recovery in the nasal region occurs in desert lizards [32]. *Dipsosaurus* [33, 34] and varanids [10, 21] were found to rely on panting to regulate head temperatures. In *Pogona vitticeps*, Tattersall and Gerlach [35] showed that tongue temperature was indeed an important aspect of the thermoregulatory strategy as its surface temperature was cooler, likely via evaporative mechanisms, than the rest of the body across a wide range of temperature and oxygen levels. Crawford [26] likewise showed that *Sauromalus* can establish a steady head-to-body temperature differential that can be abolished by taping the lizard’s mouth shut, and reestablished upon the removal of the tape. This research actually measured brain temperature, just behind the pineal organ, indicating brain cooling was occurring without the presence of a heat-exchanging rete mirabile, such as those found in birds and mammals, indicating only the use of the venous system to influence brain temperature. The oral region, on the other hand, was not found to play an active role in evaporation in rattlesnakes, indicating differing emphases of sites of thermal exchange within squamates [30]. The orbital region of squamates has unfortunately received little interest from physiologists. Yet, a few studies have made some observations on the role of the orbital region. Tattersall and Gerlach [35] recorded eye temperatures and showed that the eye is routinely cooler than the body, head, and nose across a range of temperatures and oxygen concentrations. A few other studies have included observations on the role of the orbit in thermoregulation. Webb et al. [36] found that the orbital sinuses were fully perfused with blood in *Gekko gekko*, which was visible through the oral mucosa. Mautz [37] found that the orbital region can be an important region of evaporative water loss in *Xantusia vigilis*, indicating an important possibility of the orbital sinuses influencing the temperature of neurosensory tissues.

Huey and Slatkin [7] used mathematical models to outline the use of thermoregulatory strategies, or, more specifically, the extent of a thermoregulatory response and its cost. As an example, they suggested that investing water in excessive evaporative cooling in desert or arid environments might be detrimental, when finding a better microhabitat would be less costly [7].

The literature [33, 34, 38–44] has expanded this hypothesis beyond the role of selective brain cooling as an active response to thermal environment to include an active role in water conservation. In essence, selective brain cooling abilities represent a mechanism primarily to protect water resources and, in more extreme conditions, influence central nervous system temperature. Yet, ectotherms likely use blood vessels in heating, cooling, and water conservation, indicating a more complex role for blood vessels in thermoregulation than what is indicated by the endotherm literature. All of this evidence implies variability in thermoregulatory responses depending on thermoregulatory and water conservation strategies which is exactly what can be found in the literature cited above, with different taxa responding to thermal stresses by using the nasal, oral, and orbital regions to differing extents.

Reptiles use blood vessels in a complex manner during thermoregulation and other behaviors, and current knowledge of these blood vessels should be expanded because the squamate literature is incomplete in terms of mapping vascular anatomy onto sites of thermal exchange. In a sense, the physiological literature has outpaced the anatomical literature in establishing the mechanistic underpinnings of the observed physiological strategies. The goals of this study are (1) to investigate vascular anatomy of squamates in general and iguanas in particular to determine blood vessels that could play a role in influencing head temperature, giving special attention to known sites of thermal exchange. These same sites of thermal exchange have been shown to influence head temperature in birds [29, 45], crocodilians [22] and mammals [46, 47]. Related goals of this study are (2) to document anatomical patterns and routes of potential blood flow from sites of thermal exchange to neurosensory tissues, (3) to detect and describe the osteological correlates [48] of these routes, and (4) to investigate variation in vascular patterns, with special attention to osteological correlates.

Squamates also serve a critical role as an outgroup to dinosaur studies, offering insights into more basal diapsid anatomy. Both birds and crocodilians are divergently apomorphic, offering challenges to phylogenetically informed soft-tissue studies of dinosaur soft tissues. Birds have enlarged eyeballs, expanded nasal vestibules, reduced maxillae [49] and fusion of other skull bones [50]. These apomorphies offer challenges to homologizing soft tissues, and squamates offer insights into the basal diapsid condition.

## Materials and Methods

**Fig 1:**
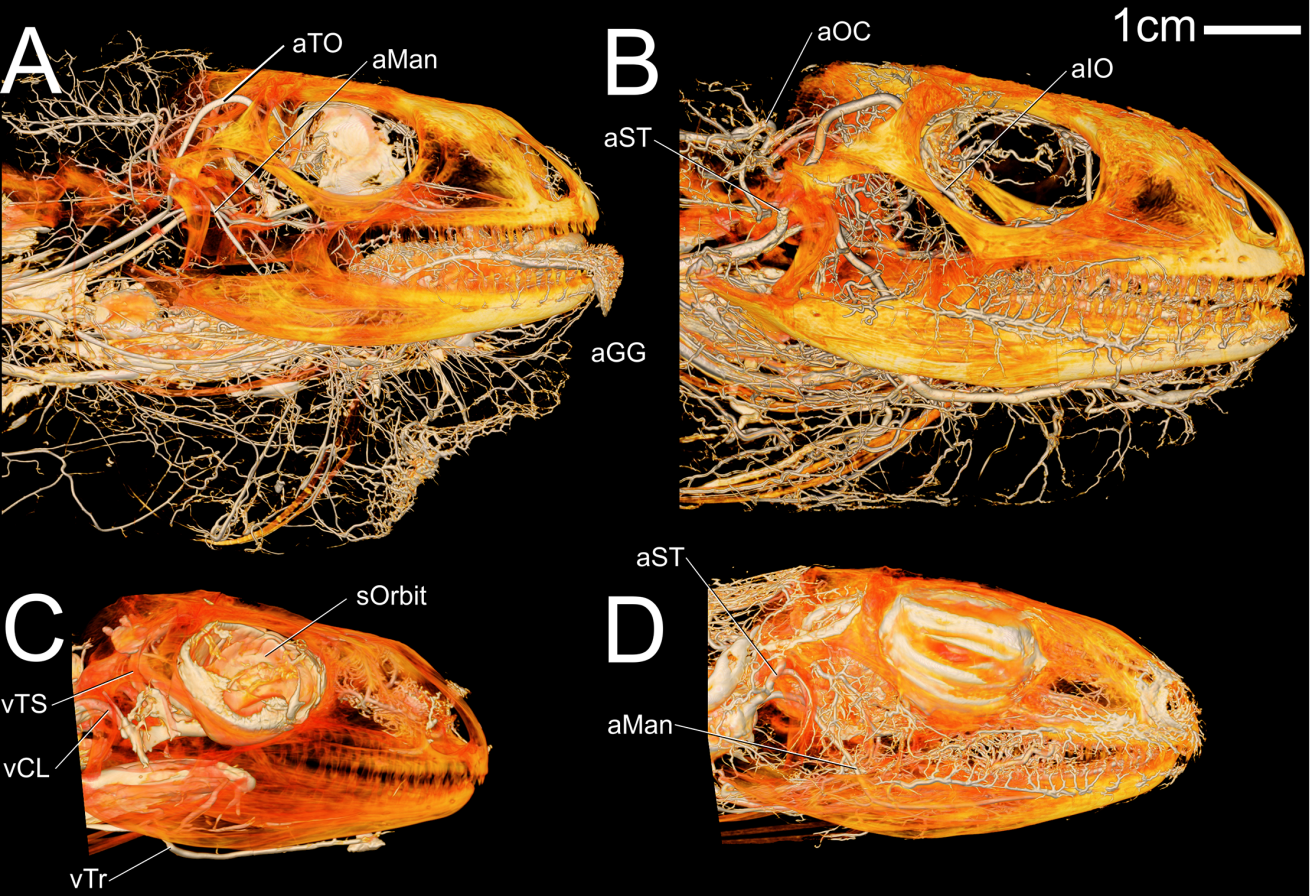
Volume renderings of four *Iguana iguana* specimens in left lateral view showing blood vessels of the head. (A) OUVC 10445 arterial injection (B) OUVC 10611 arterial and venous injection (C) OUVC 10612 venous injection (D) OUVC 10603 arterial injection.

**Fig 2:**
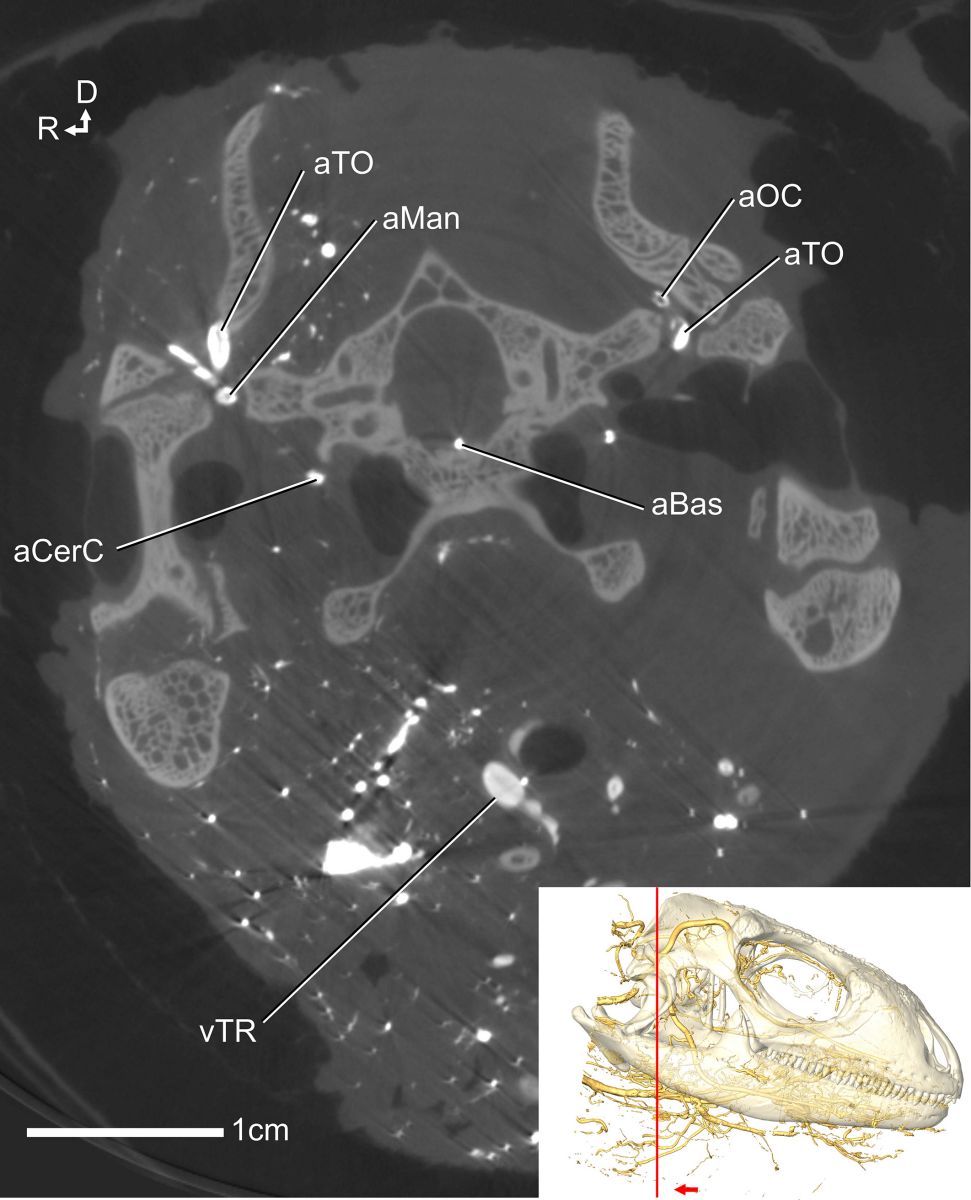
CT slice of *Iguana iguana* OUVC 10611 showing arteries and veins using the differential contrast dual vascular injection technique. Veins were injected with the solution containing less barium, resulting in a less bright region in the CT scan slice, see vTR. The arteries were injected with a greater concentration of barium, resulting in a brighter region in the CT scan slice, see aTO. D indicates dorsal and R indicates right side. Inset showing transparent skull and vascular isosurface rendering indicates slice location and orientation.

Five cadaveric specimens of *Iguana iguana* were obtained from the Miami Metro Zoo and were studied using vascular injection, CT scanning, and gross dissection. No live animals were used in this study, and no animals were euthanized expressly for use in this study. The iguana specimens were available as salvage specimens euthanized as part of a feral pest eradication program by the Miami Metro Zoo completely independently of our studies, and were provided as frozen carcasses (after they had been necropsied at the zoo) in May 2007 by Frank Ridgley, DVM, Conservation and Research Manager at the Miami Metro Zoo. The iguanas were humanely euthanized by a licensed veterinarian with an intravenous injection in the precaval vein of Euthasol euthanasia solution. These cadaveric salvage specimens were accessioned into the Ohio University Vertebrate Collection (OUVC) under the terms of Permit 14–2762 issued by the Ohio Division of Wildlife. The studied specimens (OUVC 10445, 10603, 10611, 10612, 10709) are permanently accessioned into the OUVC and are accessible to students and scholars. All specimens were frozen for variable periods of time prior to analysis and subsequently thawed prior to injection. Each specimen was scanned prior to vascular injection at the Ohio University MicroCT Scanning Facility (OUμCT) on a GE eXplore Locus in vivo Small Animal MicroCT scanner at 45 and 90μm slice thicknesses, 80kV, 450 μA (Fig 1). The carotid arteries and internal jugular veins were cannulated with a 20-gauge cannula (Becton Dickinson and Co., Franklin Lakes, NJ) and injected with a solution of colored latex (Ward’s, Rochester, NY) and barium (E-Z-EM, Westbury, NY) using the Differential-Contrast Dual-Vascular Injection (DCDVI, Fig 2) method described by Holliday et al. [51]. To better understand the arterial or venous system, one specimen was injected via the carotid artery or internal jugular vein exclusively, with the remaining specimens injected via both the carotid arteries and internal jugular vein concomitantly to facilitate DCDVI. All CT datasets are available from the Dryad database (http://dx.doi.org/10.5061/dryad.27m63).

A post-injection scan at the same settings as the first CT scan was acquired to allow the registration of the skull and soft-tissue anatomy onto the post-injection data. This sequence is necessary because the barium in the latex solution is denser than bone, which introduces some CT artifacts (e.g., beam hardening, streaking) into the data. The blood vessels course close to and within bones, creating a situation where it is nearly impossible to separate the bony and vascular signals, resulting in a skull surface that would be marred by vascular artifacts. This two-step scanning procedure alleviates these issues, resulting in a high-quality hard-tissue (e.g., skull) dataset and a high-quality vascular dataset.

Digital segmentation was completed using Avizo 7 (FEI Visualization Sciences Group, Burlington, MA) on a Dell T3400 Workstation with 8GB of RAM and an nVidia Quadro FX 4600 video card running Microsoft Windows 7 Enterprise. As noted, after data processing and analysis, the segmented tissues from the pre-injection dataset were registered to the post-injection dataset, resulting in an artifact-free skull placed in association with the injected blood vessels. This approach gives the clearest picture of the relationships between the blood vessels and bone such that they can be viewed together, in isolation, with the skull transparent, etc. Segmented blood vessels were then imported into Maya (Autodesk, San Rafael, CA) from Avizo. Maya was used to overlay 3D surfaces representing the blood vessels, using the segmented blood vessels from Avizo as templates. Using this process, the CT datasets from different iguana specimens were composited into a single model within Maya, allowing the strengths of each dataset to be represented and a more complete model generated. The outcome of this process was the generation of a diagrammatic and interactive 3D PDF digital illustration (S1 File) that allows detailed manipulation by the user/reader. The 3D PDF is considered to be an integral part of the publication and it is recommended that the reader download the 3D PDF and open it alongside the publication. The figure views are saved as “Views” in the model, along the left side. After opening the 3D PDF, click the skull in the center of the page to activate the 3D model. Now, the model in the 3D PDF can be freely rotated and zoomed, structures can be made visible, invisible, or transparent, and the vessels can be identified simply by clicking on them and reading the bolded name at left in the Model Tree.

After CT scanning and segmentation was complete, each specimen was dissected to verify the digital results. Multiple dried skulls of *Ctenosaura pectinata* (black iguana) and *Iguana iguana* (green iguana) were observed to confirm and record vascular osteological correlates [48]. Vascular nomenclature is variable in the literature, making the choice of a “standard” difficult. Oelrich’s [52] influential nomenclature was reviewed due to its prevalence in the literature, but the terminology of Sedlmayr [50] was applied, with some modifications, to cephalic blood vessels to create a common nomenclature with birds and crocodilians. Blood vessels will be described in greatest detail within three specific regions of the head, termed sites of thermal exchange, consisting of the nasal, oral, and orbital regions.

## Results

### Major Vessels of the Head

#### Overview

The major arteries supplying the head are ultimately branches of the short common carotid artery that bifurcates into the internal and external carotid arteries near the heart. The larger branches of the external carotid artery will be reported, but will not receive exhaustive descriptions as they supply the ventral tissues of the head (e.g., tongue) that are less involved in central nervous system thermoregulation. The branches of the internal carotid arteries will be reported in more detail as they supply the dorsal tissues of the head. The large vein draining the head, the lateral head vein, receives tributaries from the orbit and encephalic veins, and forms the internal jugular vein.

#### Carotid arteries (Figs 3, 4, 5, 6 and 7)

**Fig 3:**
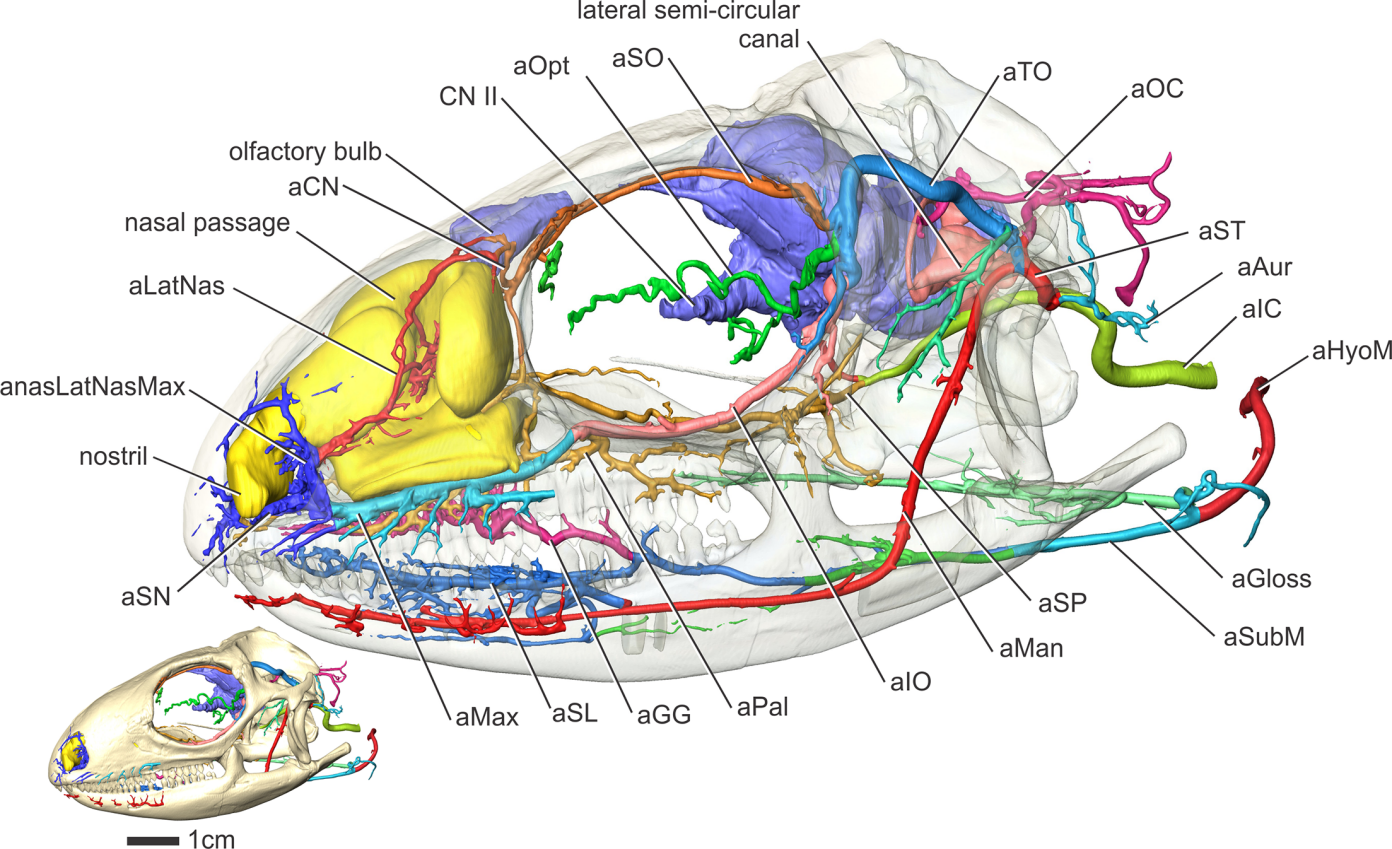
*Iguana iguana* (OUVC 10603) in left lateral view showing arteries of the head. Inset with solid skull indicates orientation of the larger image showing blood vessels and a semitransparent skull. Other soft tissues (olfactory bulbs, lateral semicircular canal) are indicated to highlight the course of aMan, aLatNas, and aSO.

**Fig 4:**
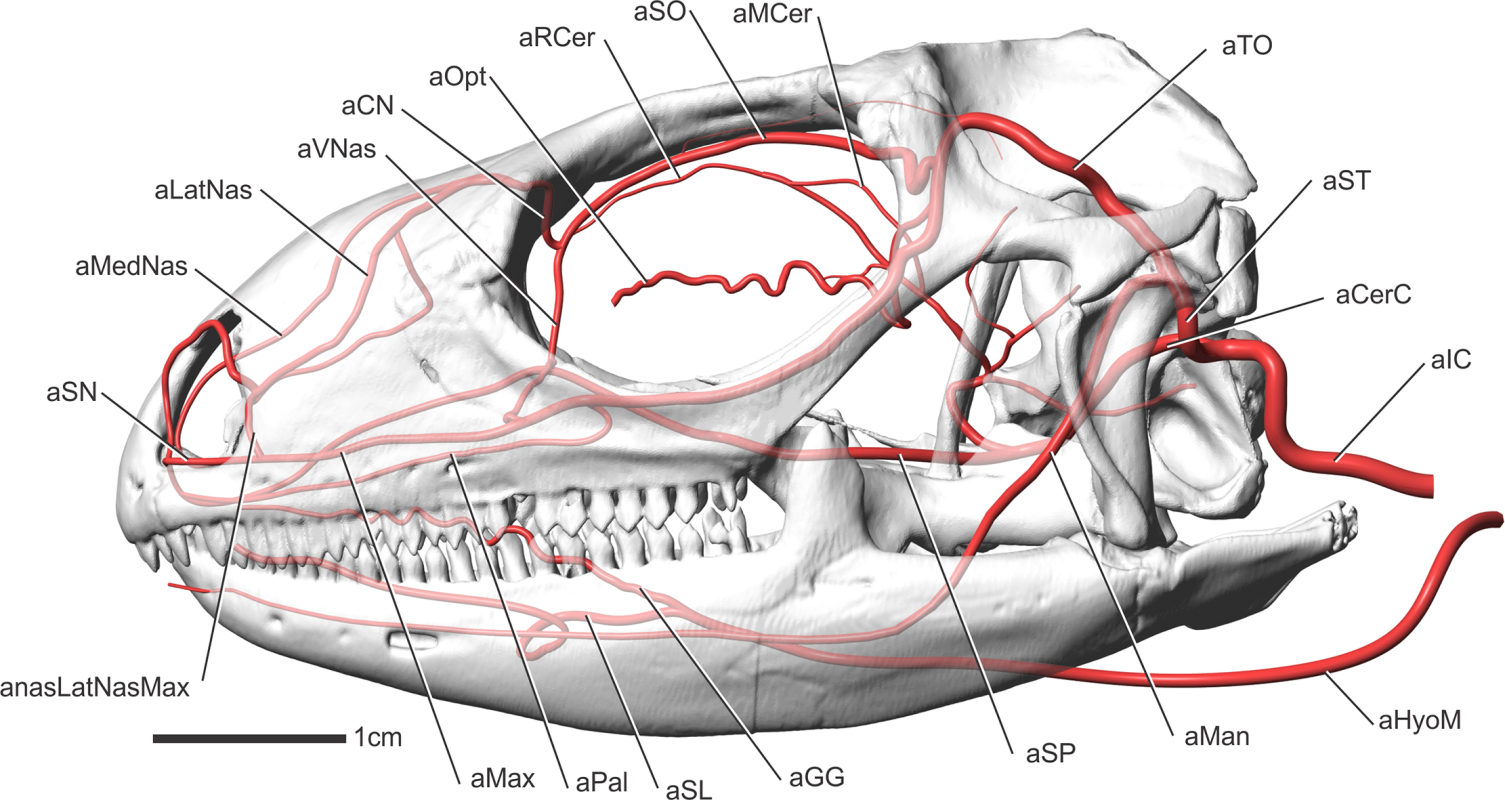
*Iguana iguana* in left lateral view showing a diagrammatic representation of the arteries of the head.

**Fig 5:**
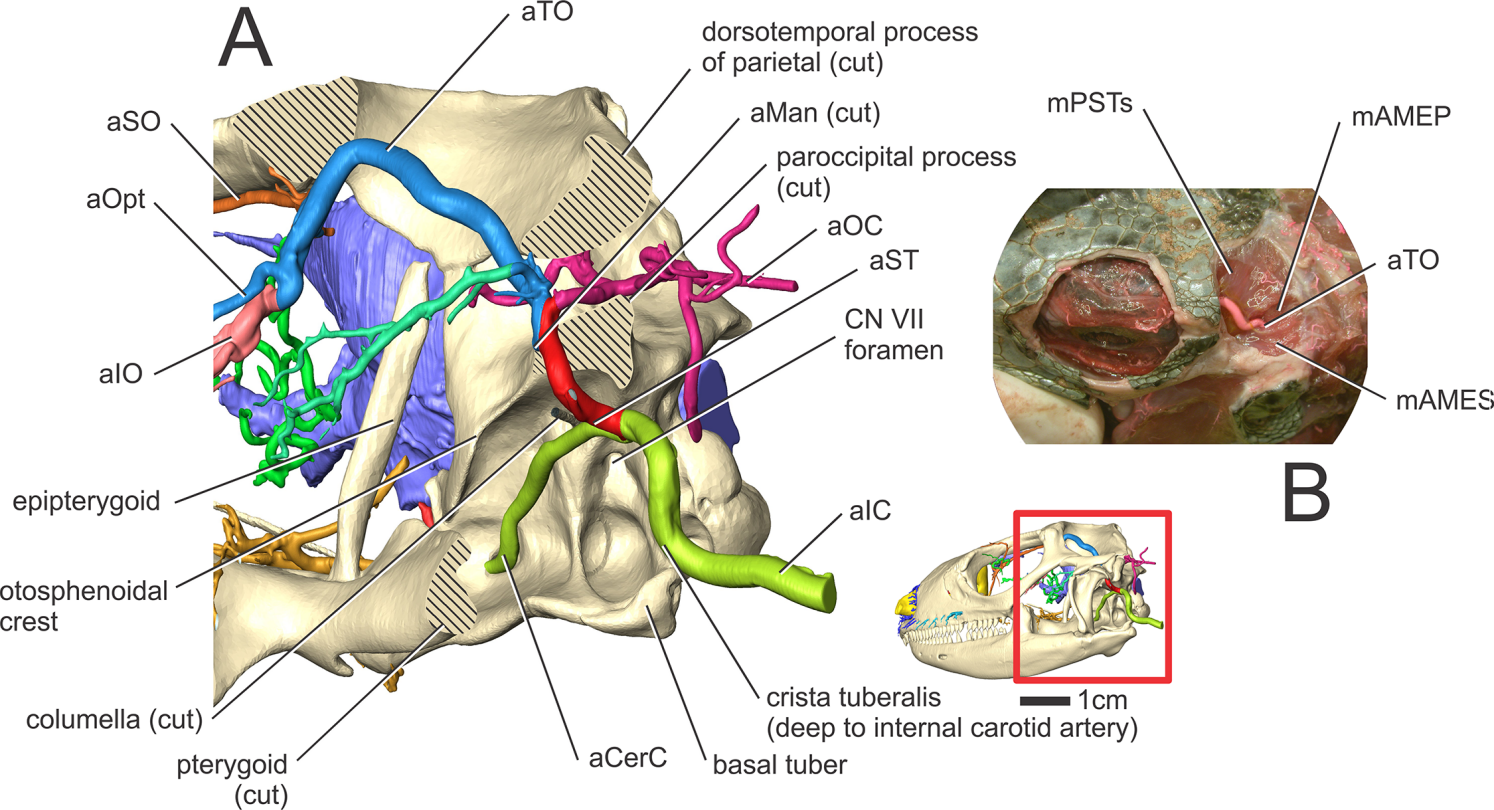
*Iguana iguana* (OUVC 10603) in left lateral view showing arteries of the head. (A) Close up of the temporal region with certain skull bones removed. The hatched regions indicate where bones were sectioned for a better view of the arterial branching patterns. The inset shows the orientation of the skull and the region of the skull in the larger image. (B) *Iguana iguana* (OUVC 10603) dissection of the dorsotemporal fossa, indicating the course of the temporoorbital artery through the jaw adductor muscles.

The internal carotid artery supplies blood to the dorsal aspect of the head, and the hyomandibular artery (Figs 3 and 4), the main branch of the short external carotid artery, supplies the tongue and surrounding tissues [52]. In ctenosaurs, the internal and external carotid arteries arise from a short common carotid artery or even directly from the aorta [52]. The external carotid artery branches into the hyomandibular and superior thyroid arteries [52]. The internal carotid (Fig 5) and hyomandibular arteries course cranially ventrolateral to the vertebrae and cervical musculature, running alongside the internal jugular vein and the vagus nerve [52]. In the iguanas studied here, the hyomandibular artery travels ventrolateral to the vagus nerve and internal carotid artery. At the level of the second cervical vertebra, the hyomandibular artery bifurcates into the glossopharyngeal and submandibular arteries [2, 52]. In the basal lepidosaur Sphenodon, O’Donoghue [2] retained the name external carotid artery after the branching of the superior thyroid artery, through the cervical region, until it branches into the hyomandibular and superficial pterygoid arteries. The superficial pterygoid arteries are small branches to the jaw muscles [2].

**Fig 6:**
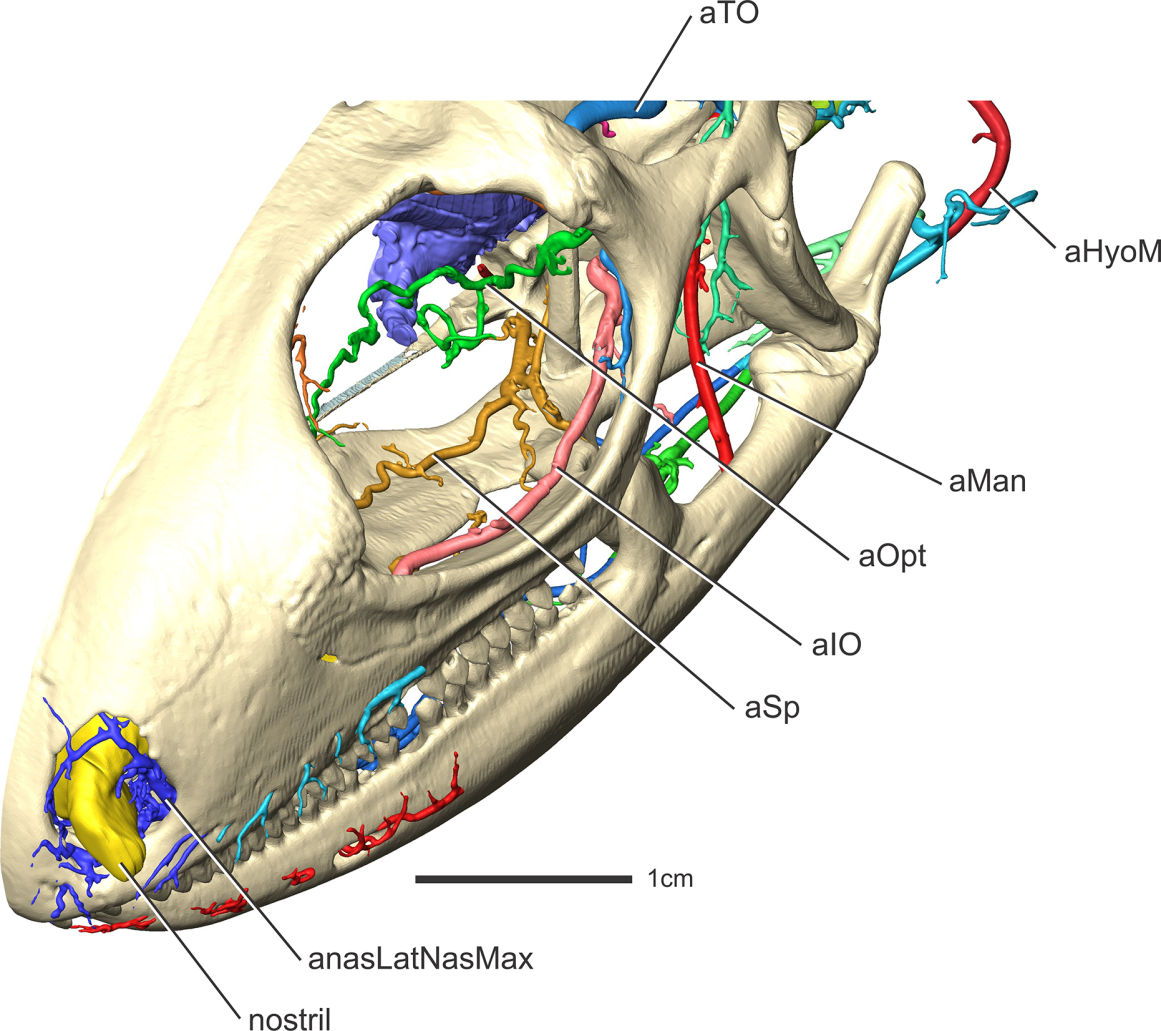
*Iguana iguana* (OUVC 10603) in left rostrodorsolateral oblique view showing the arterial patterns found on the ventral aspect of the orbital region.

**Fig 7:**
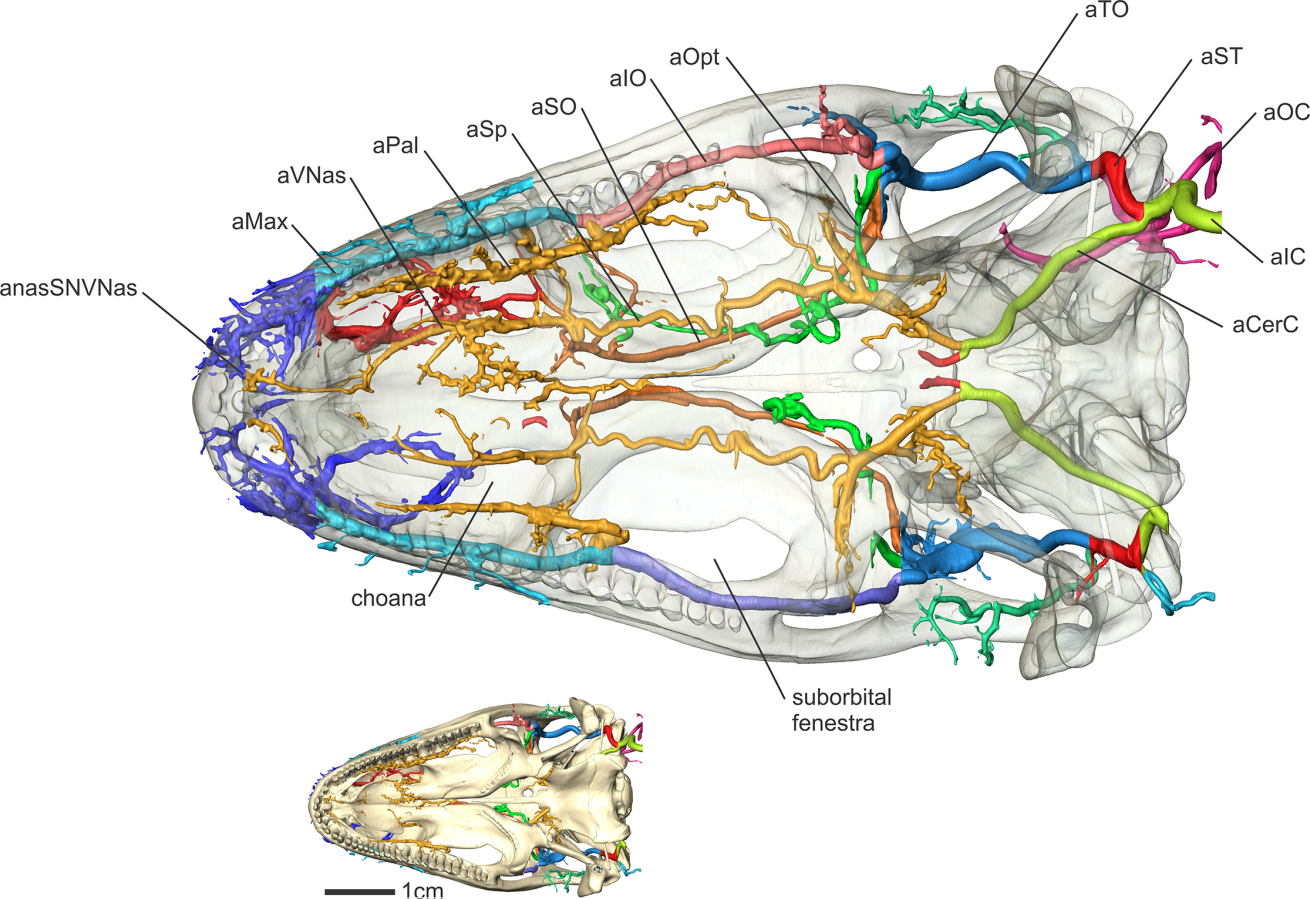
*Iguana iguana* (OUVC 10603) in ventral view showing the arteries of the palate. Inset with solid skull indicates orientation of larger image.

#### Hyomandibular artery (Figs 3, 4 and 6)

The hyomandibular artery courses rostrolaterally just caudal to the retroarticular process (Fig 6). Just ventral to the angular process, it bifurcates into the glossopharyngeal and submandibular arteries. Rostrally, the submandibular artery bifurcates into the musculomandibular and genioglossus arteries [52]. The genioglossus artery supplies the tongue [52].

#### Internal carotid artery (Figs 3, 4, 5, 7 and 8)

**Fig 8:**
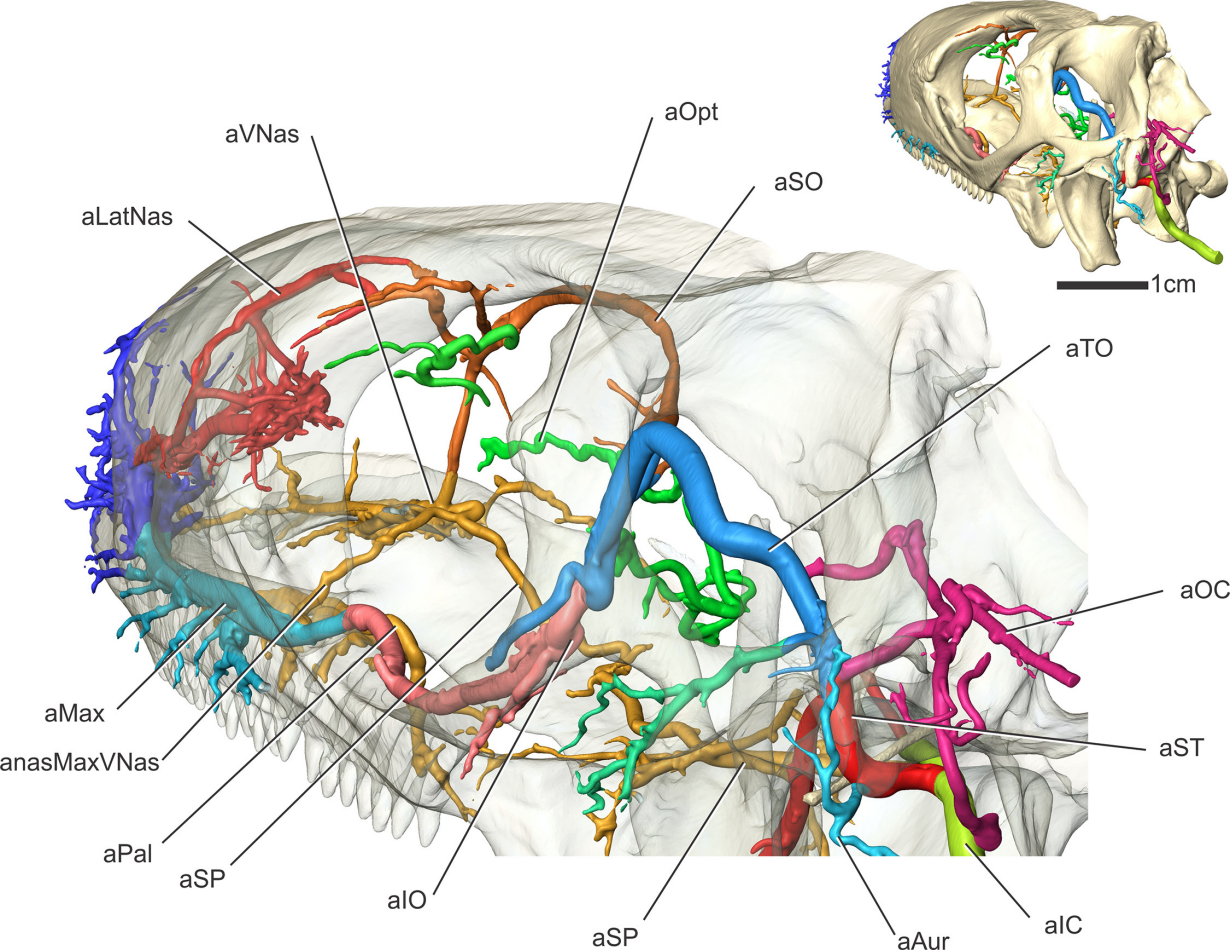
*Iguana iguana* (OUVC 10603) in left caudodorsolateral oblique view showing the arterial branching patterns in the temporal and orbital regions. Inset with solid skull indicates the orientation of the larger image.

**Fig 9:**
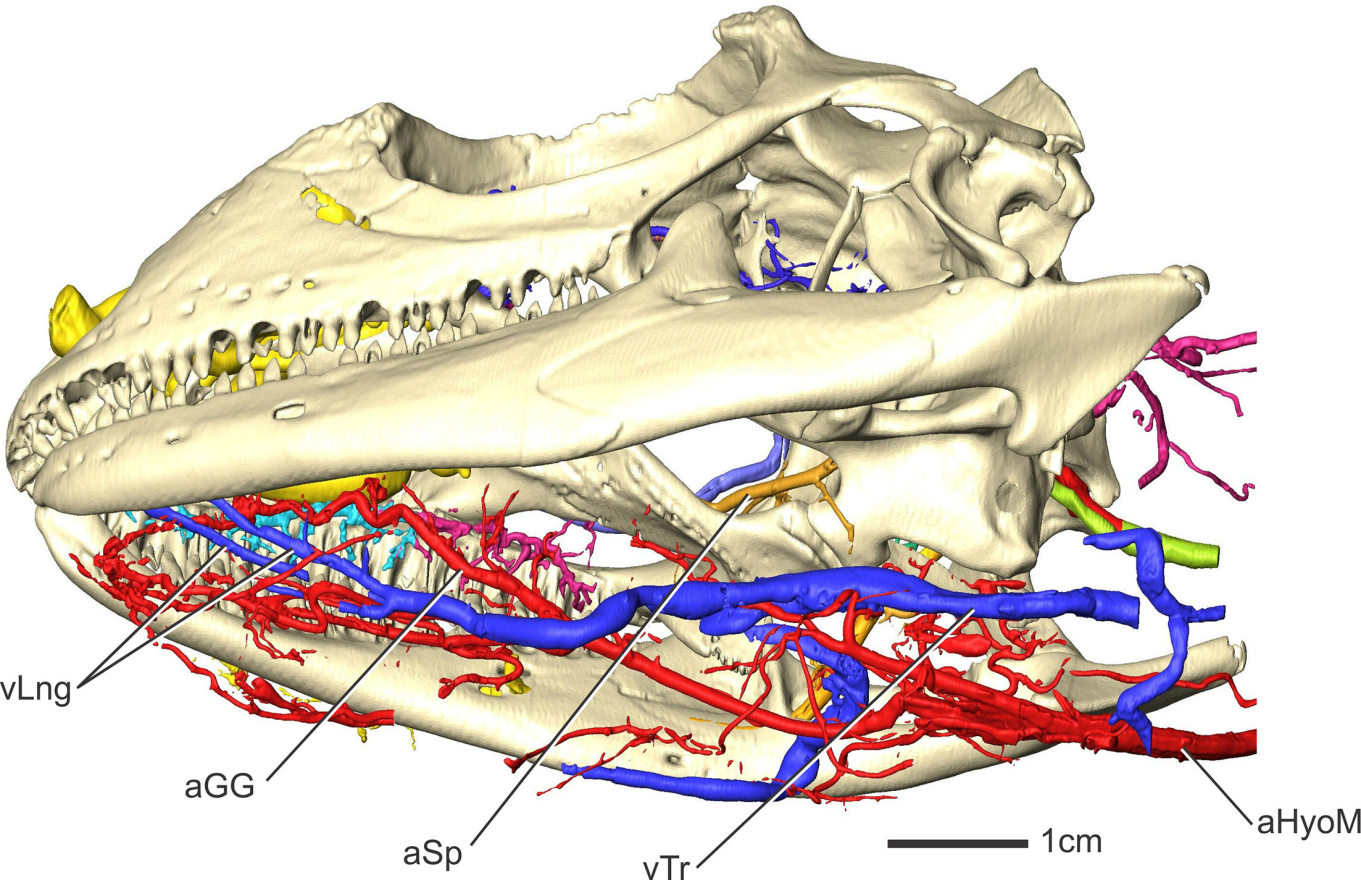
*Iguana iguana* (OUVC 10611) in left ventrolateral view showing the arteries (red) and veins (blue) along the ventral aspect of the head. These veins drain the tongue and bypass pathways that lead to neurosensory tissues.

Along the rostral border of the first cervical vertebra, just caudal to the basal tuber, the internal carotid artery makes a dorsally directed turn along the caudal aspect of the middle ear epithelium (Fig 5). The artery then courses parallel to the crista tubaris, along its lateral aspect (Figs 7 and 8). Just ventral to the paroccipital process and rostrodorsolateral to the facial nerve (CN VII) foramen, the internal carotid gives off the stapedial artery and becomes the cerebral carotid artery. In ctenosaurs, this bifurcation occurred at the level of the third cervical vertebra [52]. The cerebral carotid artery was found to pass ventral to the fenestra ovalis and columella, just lateral to the dorsal aspect of the crista interfenestralis. The cerebral carotid then passes closely apposed to the basisphenoid, ventral to the otosphenoidal crest, where it enters the cerebral carotid canal. The cerebral carotid artery courses rostrally within the carotid canal, where it gives off the sphenopalatine artery (Fig 9) and then turns dorsally into the sella turcica to supply the brain. The sphenopalatine artery (nasopalatine artery of Burda [6]), courses rostrally, through the vidian canal, to exit dorsolateral to the crista trabecularis, then passes medial to the epipterygoid and passes rostrally onto the dorsal surface of the pterygoid.

#### Stapedial artery and its branches (Figs 3, 4, 5, 7 and 8)

The other branch of the internal carotid artery, the stapedial artery, courses dorsal to the columella, then medial to the condyle of the quadrate bone. The stapedial artery then gives off the auricular artery to the tympanum. The stapedial artery then passes rostrolateral to the paroccipital process and ventral to the dorsotemporal process of the parietal bone. After passing rostrally into the dorsotemporal fossa, the stapedial artery becomes the temporoorbital artery (temporal artery of Oelrich [52]). Just rostral to the paroccipital process, the temporoorbital artery gives off the occipital artery along its caudal border. The occipital artery is a large branch from the temporoorbital artery that courses dorsally along the rostrolateral aspect of the paroccipital process, then curves dorsally along the caudal aspect of the dorsotemporal fossa. Here the occipital artery ramifies into a large plexus, just caudal to the parietal, to supply the cervical musculature. Along the rostral part of the temporoorbital artery, two arteries branch off. The dorsal branch supplies the jaw adductor musculature, and the ventral branch is the mandibular artery. The mandibular artery branches off from the temporoorbital artery at the level of the lateral semicircular canal (Figs 3 and 5). The mandibular artery then courses ventrally along the medial crest of the quadrate, then passes along the caudal border of the m. pseudotemporalis superficialis between the m. adductor mandibulae internus and m. adductor mandibulae profundus [53]. The mandibular artery then passes lateral to the pterygoid, enters the mandibular foramen (where it is renamed the intramandibular artery) and courses rostrally within the mandibular (ventral alveolar) canal. After giving off the mandibular artery, the temporoorbital artery courses dorsally through the caudal aspect of the laterotemporal fossa, then curves rostroventrolaterally along the medial aspect of the frontal-postfrontal-postorbital articulation, where it passes into the dorsotemporal fossa. This course creates an inverted “U” shape (Fig 5) between the m. pseudotemporalis superficialis and the m. adductor mandibulae externus [53] that is just deep to the skin (Figs 5 and 8). The temporoorbital artery passes medial to m. adductor mandibulae externus and lateral to m. pseudotemporalis superficialis [52, 53]. Medial to the frontal-postfrontal articulation, the temporal artery ramifies into three blood vessels that supply the orbital region.

#### Lateral head vein (Figs 10, 11 and 12)

**Fig 10:**
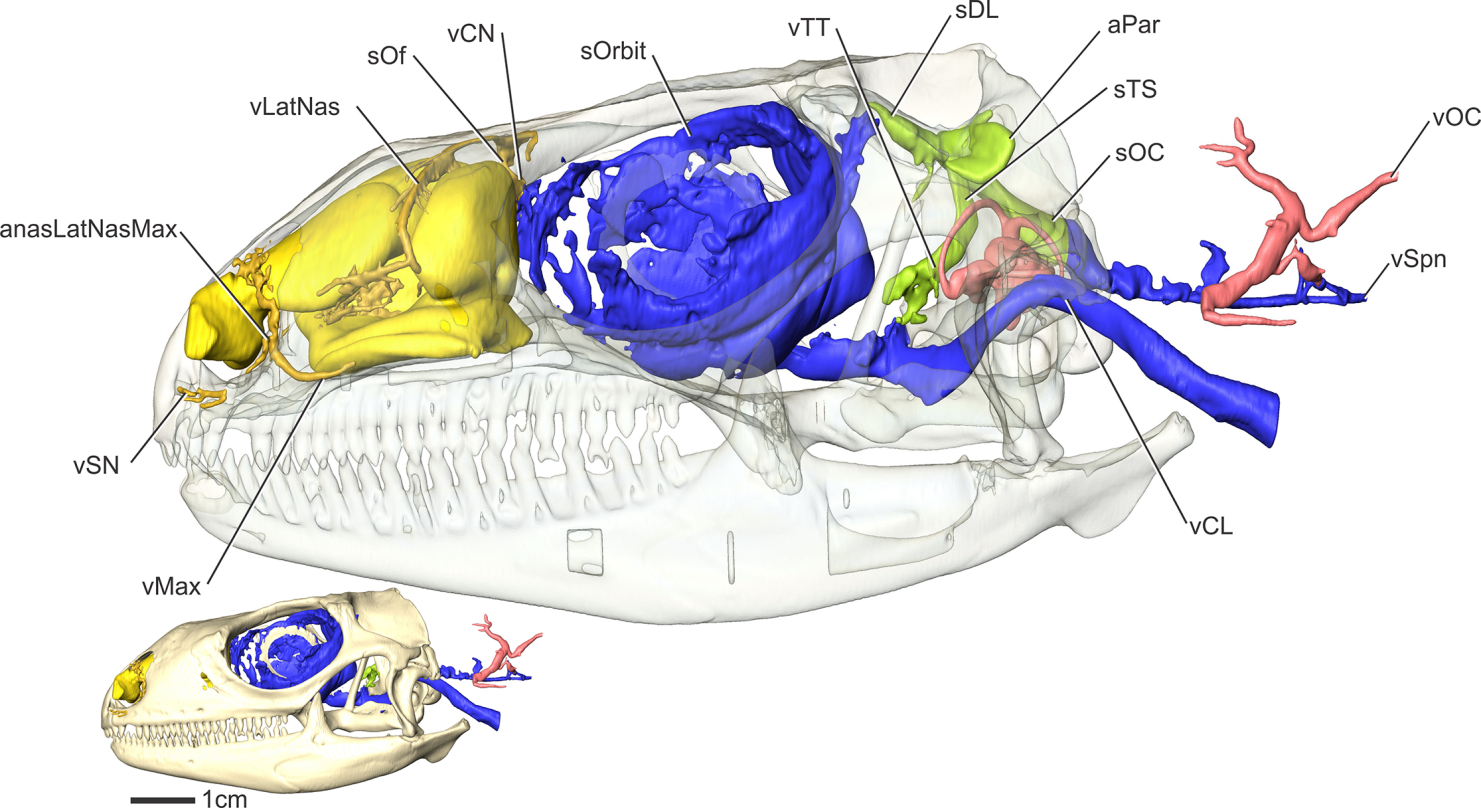
*Iguana iguana* (OUVC 10612) in left lateral view showing veins of the head. Inset image with solid skull indicates the orientation of the larger image with a semitransparent skull.

**Fig 11:**
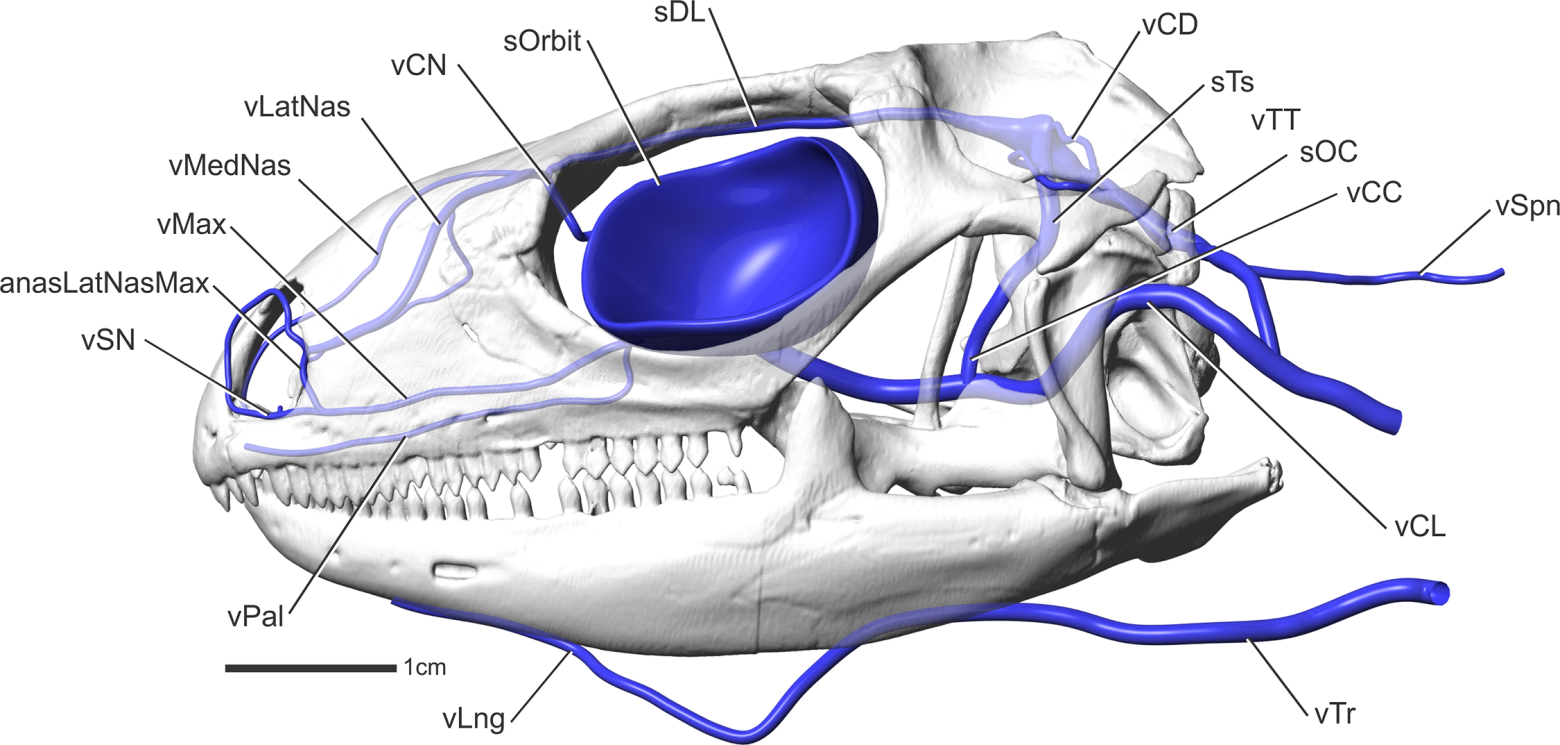
*Iguana iguana* in left lateral view showing a diagrammatic representation of the veins of the head.

**Fig 12:**
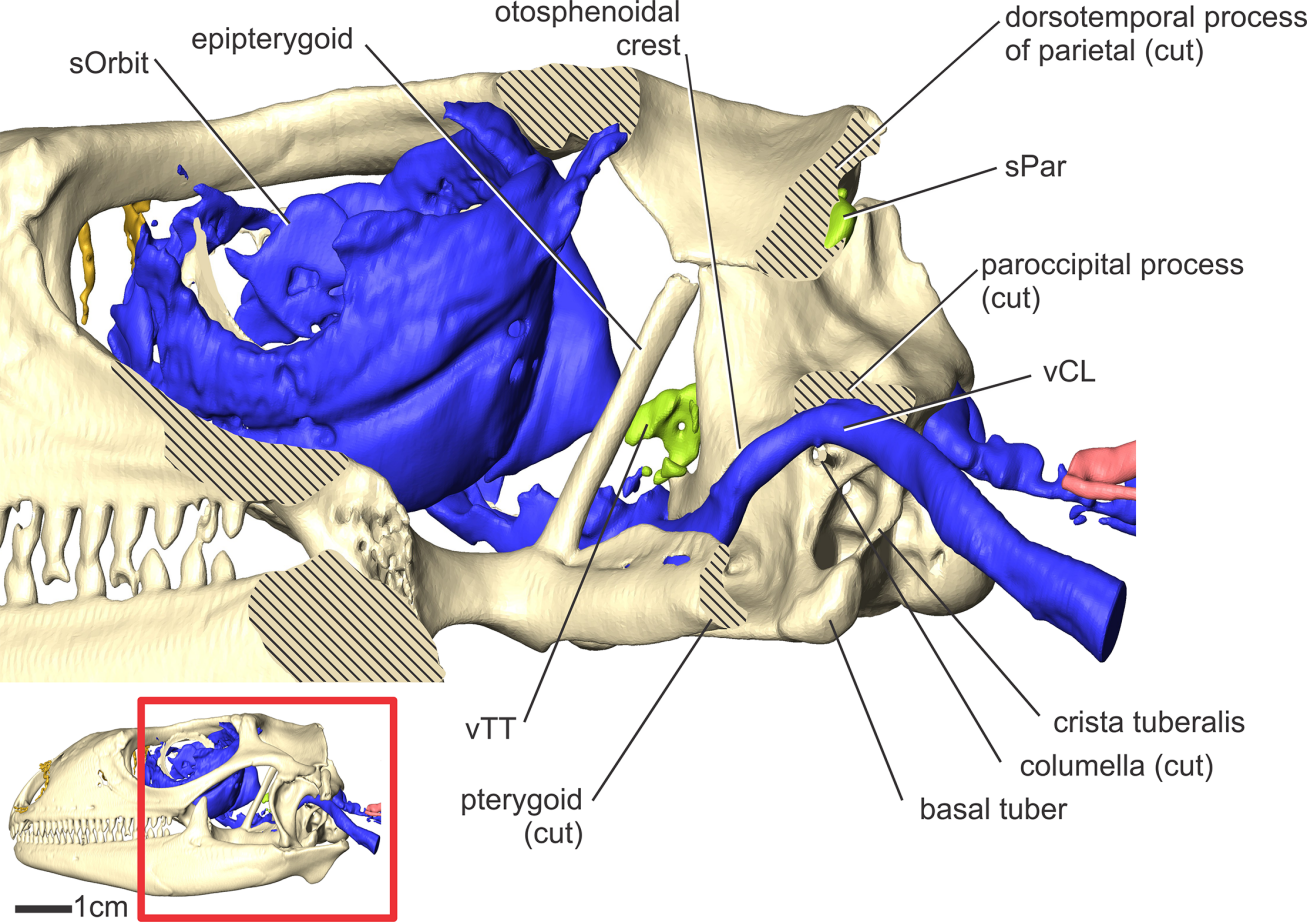
*Iguana iguana* (OUVC 10612) in left lateral view showing the veins of the head. The hatched regions indicate where bones were sectioned to have better view of the venous draining patterns. Several bones and bony features are named in the figure for reference. The inset shows the orientation of the skull and the region of the skull in the larger image.

The nomenclature for veins in the heads of reptiles has had a long and complicated past. The lateral head vein is no exception [54, 55]. Bruner [1] called this vein the internal jugular vein in Lacerta agilis. Rieppel [54] offered an appropriate explanation for the use of the term lateral head vein in snakes, citing that this vein does not exit the skull with cranial nerves IX, X, and XI [1, 52]. In Sphenodon, a vein does exit the skull with the vagus nerve and is called the posterior cerebral [2] or posterior cephalic vein [3], but this is clearly primarily an intracranial vessel whereas the lateral head vein is typically regarded as being primarily extracranial. Rieppel’s [54] terminology will be followed here. In iguanas, the lateral head vein begins in the caudoventral part of the orbit as a condensation of the orbital sinus, dorsomedial to the m. tensor periorbitae and lateral to the parasphenoid rostrum (Figs 10 and 11). The vein then courses caudally just dorsal to the basipterygoid process and medial to the epipterygoid bone where the lateral head vein then receives the hypophyseal vein [1, 52] on its medial side and the transversotrigeminal vein (middle cerebral vein of some authors) on its dorsolateral side, ventral to the trigeminal foramen [1, 52]. The lateral head vein then makes a dorsally directed turn along the prootic bone, coursing caudally, closely following the caudoventral contours of the otosphenoidal crest (Fig 12). The lateral head vein then courses caudally, medial to the quadrate, to pass into the middle ear region, where the vein is covered ventrally by the epithelium of the middle-ear sac. The lateral head vein then courses through the cranioquadrate passage between the quadrate and braincase, then passes dorsal to the columella and the stapedial artery. The vein continues to course caudolaterally to pass ventral to the paroccipital process, medial to m. depressor mandibulae, ultimately to become the internal jugular vein, which then runs toward the heart adjacent to the internal carotid artery and the vagus nerve [2].

### Orbital Region

#### Overview

The arteries that supply the orbital region branch off from the temporoorbital, specifically the infraorbital, supraorbital, and ophthalmotemporal arteries. The infraorbital artery forms the maxillary artery after it enters the maxilla from the orbit. The supraorbital artery supplies tissues of the orbit, yet is also the major supplier of blood to the nasal region. The ophthalmotemporal artery supplies primarily the eyeball. The major vein of the orbit, the orbital sinus, receives the nasal vein from the nasal region, the maxillary vein from the oral region, and blood from the eyeball. The orbital sinus then coalesces into the lateral head vein. Because the orbital sinus receives blood from the nasal and oral regions and the eyeball, essentially all three sites of thermal exchange, the blood contained within it likely has the ability to regulate the temperature of the eyeball.

#### Supraorbital artery (Figs 3, 4, 7 and 8)

Along the caudodorsolateral aspect of the orbit and the rostral surface of the m. pseudotemporalis superficialis, the temporoorbital artery ramifies (Figs 3 and 4) into three major branches [52]. The dorsal most is what Oelrich [52] called the frontal artery, more often known as the supraorbital artery [2, 56], which will be followed here as this artery is likely homologous to the supraorbital arteries of birds and crocodilians. The supraorbital artery courses rostromedially along the ventral surface of the frontal along with the ophthalmic branch of the trigeminal nerve [52] and sends many smaller-diameter branches laterally along the ventral surface of the frontal. The supraorbital artery also anastomoses with the olfactory artery, the rostralmost branch of the rostral cerebral artery [6], indicating that the cerebral arteries can also supply the nasal cavity in squamates, albeit to a lesser degree when compared to archosaurs. Burda [6] reported this condition in Lacerta embryos, but stated that the olfactory arteries atrophy to a varying degree in lizards. In iguanas, the olfactory artery was clearly found to anastomose with the supraorbital and ventral nasal arteries rostroventral to the olfactory bulb. Along the rostral aspect of the orbit, the supraorbital artery gives off an artery (the rostral orbital artery; Oelrich [52]) into the Harderian gland. Another branch from the supraorbital artery supplies the dorsal and ventral oblique muscles, similar to ctenosaurs [52]. The supraorbital artery then bifurcates into two branches along the caudodorsal aspect of the orbitonasal membrane (Figs 4 and 8). The ventrally directed branch is the ventral nasal artery, which anastomoses with the sphenopalatine artery. The rostrodorsally directed branch is the common nasal artery (dorsal nasal artery of Oelrich, [52]).

#### Ophthalmotemporal artery (Figs 3, 4, 5, 6, 7 and 8)

The second branch from the temporoorbital artery is the ophthalmotemporal artery. This artery has been named both the superior orbital artery [52] and ophthalmic artery [2] in the past. To clear some confusion, this artery will be called the ophthalmotemporal artery, as in birds [29, 45] and crocodilians [50]. The ophthalmotemporal artery courses medially along the caudal aspect of the orbit (Fig 6), then between the m. dorsal rectus and m. lateral rectus, passes dorsal to the optic nerve, and then rostrally along the medial aspect of the eyeball [52]. The ophthalmotemporal artery then pierces the sclera to supply the eyeball. Oelrich [52] described a branch that supplied the conjunctival membrane and the lacrimal gland, but in iguanas this artery branched directly from the temporoorbital artery rather than the ophthalmotemporal artery. The ophthalmotemporal artery anastomoses with the internal ophthalmic artery just dorsomedial to the optic nerve, similar to the condition described by Oelrich [52] and Burda [6]. The internal ophthalmic artery is a branch of the rostral cerebral artery [6, 52], connecting the intracranial and extracranial vasculature, a similar condition being found in birds [29] and crocodilians [50].

#### Infraorbital artery (Figs 3, 5, 6, 7 and 8)

The third and largest branch of the temporoorbital artery is the infraorbital artery [56] (inferior orbital artery of Oelrich [52]). This artery courses rostrally along the ventrolateral aspect of the eyeball, between the orbital fascia and the m. tensor periorbitae, and sends branches laterally to the conjunctival glands. Ventral to the eyeball, the infraorbital artery courses with the maxillary division of the trigeminal nerve. The infraorbital artery then courses rostrally towards the infraorbital foramen in the maxilla [52]. Caudal to this foramen, the infraorbital artery gives off the ventrally directed palatine artery (Figs 3 and 7). This artery curves ventrally around the caudal aspect of the maxillary process, traversing the suborbital fenestra to supply the salivary glands along the medial aspect of the tooth row [52].

In ctenosaurs, Oelrich [52] described an artery branching from both the infraorbital and supraorbital arteries to supply the Harderian gland. In iguanas, this rostral orbital branch from the supraorbital artery was not found to anastomose with the infraorbital artery. What Oelrich [52] described as the anterior orbital branch of the infraorbital artery supplies a rather large conjunctival gland along the rostroventral aspect of the orbit in iguanas. Rostral to giving off the rostral orbital artery, the infraorbital sends a branch that accompanies the lacrimal duct [52]. The infraorbital artery then enters the infraorbital foramen within the maxilla and changes its name to the maxillary artery [52]. Within the maxilla, the maxillary artery sends a small branch that anastomoses with the sphenopalatine artery, just caudal to both the nasal cavity and the anastomosis of the ventral nasal and sphenopalatine arteries (Fig 8). This artery passes along the rostral surface of the maxillary process, enters a canal in the palatine and courses dorsomedially. The artery then exits the canal on the dorsal surface of the palatine bone through the palatine foramen [52], just rostral to the articulation between the maxilla and the palatine, and anastomoses with the sphenopalatine artery.

#### Sphenopalatine artery (Figs 3, 4, 6, 7, 8 and 9)

The sphenopalatine artery [52] (nasopalatine artery of Burda [6]) is a rostrally directed branch from the cerebral carotid artery. This artery courses through the Vidian canal with the palatine branch of the facial nerve (CN VII), and exits the basisphenoid dorsal to the crista trabecularis [52]. In birds [29] and crocodilians [50], the sphenopalatine artery shares a similar branching pattern and likewise runs with the palatine branch of CN VII and shares similar anastomotic connections with the nasal arteries. The largest branch of the sphenopalatine artery in the iguana sample, the epipterygoid artery [52], runs dorsally along the medial aspect of the epipterygoid bone. Rostral to the branching of the epipterygoid artery, the sphenopalatine artery sends ventrally directed branches that supply the mucosa of the piriform [52] (interpterygoid) recess, along the medial border of the pterygoid bones. The sphenopalatine artery then courses rostrally along the floor of the orbit, ventral to the orbital membrane and m. tensor periorbitae, sending branches ventrally, medially, and laterally. The ventrally directed branches supply the region of the palate rostral to the pterygoid teeth, the caudal aspect of the choana, and the large glandular area located on the midline of the vomer [52]. The laterally directed branches anastomose with the palatine artery just before it curves ventrally around the maxilla. In ctenosaurs [52], this artery branches off from the ventral nasal artery. Just caudal to the orbitonasal membrane, the sphenopalatine artery anastomoses with the ventral nasal artery along its dorsal surface (Fig 8). The sphenopalatine artery then passes through a foramen in the nasal cartilage to pass ventral to the solum nasi of the cartilaginous nasal capsule as the continuation of the ventral nasal artery. The ventral nasal artery then courses along the dorsal aspect of the vomer to supply the vomeronasal organ, after which it anastomoses rostrally with branches of the subnarial artery within the premaxilla [52].

#### Veins of the orbital region (Figs 10, 11 and 12)

The veins of the orbital region were described for four taxa by Bruner [1]. The veins within the orbit of iguanas largely agree with Bruner’s [1] account, with the largest vein being the orbital sinus. The orbital sinus was found to envelop the eyeball along its medial aspect. The orbital sinus did not pass dorsally beyond the ophthalmic branch of the trigeminal nerve, the tendon of the muscle of the nictitating membrane, or the trochlear nerve [1, 2]. Rostrally, the orbital sinus enveloped the dorsal and ventral oblique muscles, as reported by Oelrich [52] for Ctenosaurus. In the rostrodorsal corner of the orbit, the orbital sinus receives the nasal vein from the nasal region. The orbital sinus passes ventral to m. ventral rectus and caudal to the lateral rectus and the bursalis muscles. The orbital sinus coalesces ventromedially along the caudal aspect of the optic nerve to form the lateral head vein along the medial side of the m. tensor periorbitae and lateral to the parasphenoid rostrum (Fig 8).

### Nasal Region

#### Overview

The arteries supplying the nasal region are branches of the nasal artery, formed by the anastomosis between the supraorbital and rostral cerebral (olfactory) arteries. The nasal artery bifurcates into the medial and lateral nasal arteries. The medial nasal artery courses along the nasal septum and enters the premaxilla. The lateral nasal artery exits the cartilaginous nasal capsule and supplies the nasal gland, then anastomoses with the maxillary artery. The veins of the nasal region closely follow the arteries of the same name, with the nasal vein draining into the orbital sinus and the dorsal longitudinal sinus, allowing blood to drain into multiple pathways. The ventrally directed branch of the nasal vein drains into the orbital sinus along the rostrodorsal aspect of the orbit, passing through the orbitonasal fissure [1]. The dorsal midline branch of the nasal vein drains into the dorsal longitudinal sinus.

#### Common nasal artery (Fig 8)

The dorsal aspect of the nasal region is supplied by the supraorbital and olfactory arteries [6]. Caudal to the orbitonasal membrane, the supraorbital artery branches into the common and ventral nasal arteries. The ventral nasal artery courses ventrally to anastomose with the sphenopalatine artery, and the common nasal artery passes in a rostrodorsal direction through the orbitonasal fissure, medial to the sphenethmoidal cartilages to enter the nasal capsule [52, 56]. Along its medial side, the supraorbital artery receives the olfactory artery from the rostral cerebral artery [6], providing the nasal cavity and olfactory bulbs with collateral blood supply [6]. The common nasal artery curves dorsolaterally around the olfactory bulb, and then branches into two arteries, the medial and lateral nasal arteries (Figs 3 and 8). The medial nasal artery runs alongside the medial nasal branch of the ophthalmic nerve. As Bruner [1] noted, the medial nasal artery runs along the nasal septum, coursing with the medial nasal vein and nerve. The lateral nasal artery courses rostrolaterally and pierces the nasal cartilages to course rostroventrally along the dorsolateral side of the nasal capsule with lateral nasal branch of the ophthalmic nerve, passing lateral to the auditis conchae. It sends a large branch into the lateral nasal gland, courses ventrolateral to the zona annularis, and ultimately anastomoses with the maxillary artery. In crocodilians, the maxillary and lateral nasal vessels anastomose, in a similar manner as squamates. A large branch of the lateral nasal artery passes through the ventral aspect of the lateral nasal gland [52] and rejoins the main lateral nasal artery along the medial side of the anastomosis between the maxillary and lateral nasal arteries.

#### Ventral nasal artery (Figs 4 and 8)

The rostral and ventral aspect of the nasal region is supplied by the ventral nasal and maxillary arteries [52]. The ventral nasal artery, which is the continuation of the sphenopalatine artery rostral to the orbit, supplies the ventral aspect of the nasal cavity [52]. This artery courses along the dorsal surface of the vomer within a groove. Along the caudomedial aspect of the choana, the ventral nasal artery sends a branch along the ventral aspect of the palatine that supplies the glandular tissues along the rostromedial aspect of the vomer. Branches of the ventral nasal artery also supply this plexus caudally. The ventral nasal artery continues along the dorsal aspect of the vomer, supplies the vomeronasal gland, and then anastomoses with the subnarial arteries rostrally.

#### Lateral nasal vein (Figs 10, 11 and 12)

The rostral course of the lateral nasal vein courses along the ventral aspect of the zona annularis [52], sending a tributary to the maxillary vein. Bruner [1] did not report the connection between the maxillary vein and the lateral nasal vein in Lacerta agilis, and described the lateral nasal vein as forming a short connection between the lateral nasal sinus and the maxillary sinus [1]. Further caudally, the lateral nasal vein runs external to the nasal capsule with the lateral nasal artery and the lateral nasal branch of the ophthalmic nerve, passing lateral to the auditus conchae and accepting tributaries from the lateral nasal gland. Along the ventral surface of the auditus conchae, the lateral nasal vein ramifies in a plexus that drains the ventral and rostroventromedial aspect of the zona annularis, the rostroventral aspect of the concha, and the space along the caudal aspect of the septomaxilla. This region is highly vascularized, and is not obvious in gross dissections whether this region contains cavernous tissue with erectile properties in Iguana, although it is likely to be present [57]. The lateral nasal vein anastomoses with the maxillary vein, forming a similar arc along the dorsal aspect of the flesh nostril, named the vena rostralis by Bruner [1], and likely drains the cavernous tissue associated with the nostril [57].

#### Medial nasal vein (Fig 11)

The medial nasal vein shares an anastomosis with the lateral nasal vein, where it then courses medially, along the rostral narial fossa. The medial nasal vein then turns medial to the nasal septum, where it courses caudodorsally along with the medial nasal artery. The medial nasal vein then receives the lateral nasal vein to form the common nasal vein.

#### Common nasal vein (Figs 10 and 11)

The common nasal vein is formed by the union of the medial and lateral nasal veins. The nasal vein has an anastomotic connection to the olfactory sinus around the olfactory bulb. The nasal vein then passes through the orbitonasal fissure, and takes a ventrally directed course to empty into the orbital sinus.

### Oral region

The oral region is supplied by multiple arteries. The infraorbital artery sends a branch through the suborbital fenestra to the medial aspect of the tooth row to supply the lateral portion of the palate. The medial portion of the palate is supplied by the sphenopalatine artery rostral to the anastomosis with the ventral nasal artery. The ventral nasal artery sends branches into the vomeronasal organ and between the vomer bones to the dorsomedial part of the palate. The veins of the oral region, the maxillary and palatine veins, drain into the orbital sinus.

#### Sphenopalatine artery (Figs 3, 4, 6, 7 and 8)

The oral region was found to be highly vascularized, with supply originating from arteries of the orbital and nasal regions. Oelrich [52] reported that the sphenopalatine artery sent branches through the pterygoid bone and supplied the palate near the region of the pterygoid teeth. This was confirmed in iguanas as the sphenopalatine artery sends branches along the dorsal surface of the piriform recess and along the basipterygoid process to supply the general region of the pterygoid caudal to the pterygoid teeth and the oral mucosa lining the caudoventral aspect of the basipterygoid region. These branches also course caudally to supply the dorsal aspect of the pharynx. Branches of the sphenopalatine artery also run ventrally around the pterygoid bone to supply the oral mucosa rostral to the pterygoid teeth.

#### Subnarial artery (Figs 3 and 4)

The subnarial artery is the continuation of the maxillary artery rostral to the nostril and supplies the rostral aspect of the palate (Fig 3) via branches that pass caudoventrally through canals in the premaxilla found at the caudolateral aspect of the nasal process. These branches from the subnarial artery supply the glandular (median vomerine gland of Bellairs [56]) region found medial to the choana, resulting in a highly vascularized and highly innervated region [52]. The sphenopalatine artery also sends a branch into this plexus/glandular region by sending a branch along the medial wall of the choana, along the lateral aspect of the palatine bone, then coursing rostrally into the glandular area.

#### Palatine artery (Figs 3, 7 and 8)

The palatine artery supplies the glands along the lateral aspect of the palate, medial to the tooth row (the lateral palatine strip of Oelrich [52]). Before entering the infraorbital foramen, the infraorbital artery sends a ventrally directed branch, the palatine artery [52] that passes along the caudal aspect of the maxillary process of the palatine bone. The palatine artery has an anastomosis with a branch of the subnarial artery that passes through the premaxilla.

#### Maxillary artery (Figs 3, 4, 7 and 8)

The maxillary artery begins at the infraorbital foramen [52] and courses rostrally though the dorsal alveolar canal. The maxillary artery sends branches laterally that exit on the lateral surface of the maxilla to supply the oral margin (Fig 1). Interestingly, the roof of this canal is transient in iguanas. The maxillary process of the palatine roofs the canal caudally whereas rostrally the canal is open dorsally and is separated from the nasal cavity only by the cartilaginous nasal capsule along the lateral side of the choana. The nasal capsule is the only tissue that separates this artery from the airstream, potentially allowing heat to be exchanged. The maxillary artery courses through the dorsal alveolar canal, and exits the canal onto the floor of the narial region [52]. Just caudal to the fleshy nostril, the maxillary artery (Fig 3) gives off a dorsally directed artery to the lateral nasal artery and continues rostrally as the subnarial artery [52]. Branches from the lateral nasal and maxillary arteries anastomose superficial to the nasal capsule and arc along the dorsal surface of the fleshy nostril, then around the medial side to anastomose with the subnarial artery. These branches supply a ring of cavernous tissue around the nostril that is likely erectile in nature found along the ventral and lateral aspects of the vestibule [57]. The subnarial artery continues rostromedially ventral to the fleshy nostril along the vomerine process of the maxilla [52]. The subnarial artery sends branches to supply the mucosa of the nasal vestibule, whereas branches that course caudodorsally ventral to the vomer supply the rostral aspect of the palate. These branches pass dorsally through a foramen in the vomer to anastomose with the ventral nasal artery, and together they supply the vomeronasal gland. The subnarial artery continues into the premaxilla, where it is accompanied by the medial nasal branch of the ophthalmic nerve [52].

#### Maxillary vein (Figs 10 and 11)

Along the caudal aspect of the premaxilla, the subnarial vein accepts the vena rostralis and continues ventral to the nostril. Caudal to the nostril, the maxillary vein receives a tributary from the lateral nasal vein and then courses caudally through the dorsal alveolar canal with the maxillary artery and nerve. Lateral to the choana, where the roof of the dorsal alveolar canal is not present, the maxillary vein receives a vein draining the concha [1]. The maxillary vein exits the infraorbital foramen and drains into the orbital sinus [1].

#### Palatine vein (Fig 11)

The palatine vein courses closely with the palatine artery. Along the rostroventral border of the premaxilla, the palatine vein courses caudally, draining the lateral aspect of the palate. At the rostral border of the suborbital fenestra, the palatine vein curves dorsally to drain into the orbital sinus.

#### Vasculature of the tongue (Fig 9)

The tongue was found to be highly vascularized and iguanids have been known to extend the engorged tongue during thermoregulation [33]. The submandibular artery supplies the tongue through the genioglossus artery [52]. The venous drainage of the tongue bypasses cephalic neurosensory tissues in iguanas, even though it is positioned directly ventral to the choana and may have cooled air from the choana aiding evaporation from the tongue. Bruner [1] noted this bypass when describing the venous drainage of the tongue as being through the tracheal vein. Templeton [33] also found that panting had little effect on body temperature and concluded that evaporative cooling of the oral cavity had no effect on brain temperature in Dipsosaurus. Dewitt [24], however, did find that panting played a role in maintaining the head 3°C cooler than the body in Dipsosaurus. Tattersall and Gerlach [35] also showed that the tongue was an important component in the thermoregulatory strategy of bearded dragons. Thus, the role of the tongue in cooling tissues of the head is still uncertain, and the results reported here show no direct anatomical mechanism to promote thermoregulation of cephalic tissues. The dorsal and ventral lingual veins [1] drain into the buccal vein to form the tracheal vein. The right and left tracheal veins unite along the midline and travel through the right side of the cervical region to join the rostral vena cava on the medial side of the internal jugular vein [1].

## Discussion

Squamates have been shown to use the circulatory system to facilitate increases or decreases in whole-body temperature by controlling peripheral blood flow [11, 12] and the subsequent heart-rate hysteresis [10, 13, 14, 15], and activating the jugular constrictor muscle [1, 4, 5, 17, 24]. These cardiovascular adjustments have been shown to be important in thermoregulatory patterns of squamates and is likely advantageous to finely control body temperatures and ultimately increase activity periods [4, 7, 15, 44, 58].

The use of the jugular constrictor muscle has received some attention in the 1960s and 1970s, but the idea has not been explored since. The role of these muscles is thought to influence head temperature by allowing the preferential *warming* of the head [4] via a counter-current heat exchange between the internal jugular and internal carotid artery [4]. More recently, studies of physiological thermoregulation have been showing that evaporative *cooling* may hold an important role in influencing head temperature, specifically within sites of thermal exchange. The demonstrated role of the oral cavity in influencing brain temperature in *Sauromalus* [17] and cooled exhaled air in *Dipsosaurus* [32] indicates a prominent role for evaporative cooling in sites of thermal exchange. The blood supply and drainage from each of these sites of thermal exchange offer the potential to move thermal energy around the head. The jugular constrictor muscle may have a role in influencing this movement of thermal energy by influencing venous drainage pathways from sites of thermal exchange around the head, or more specifically, around neurosensory tissues of the central nervous system.

**Fig 13:**
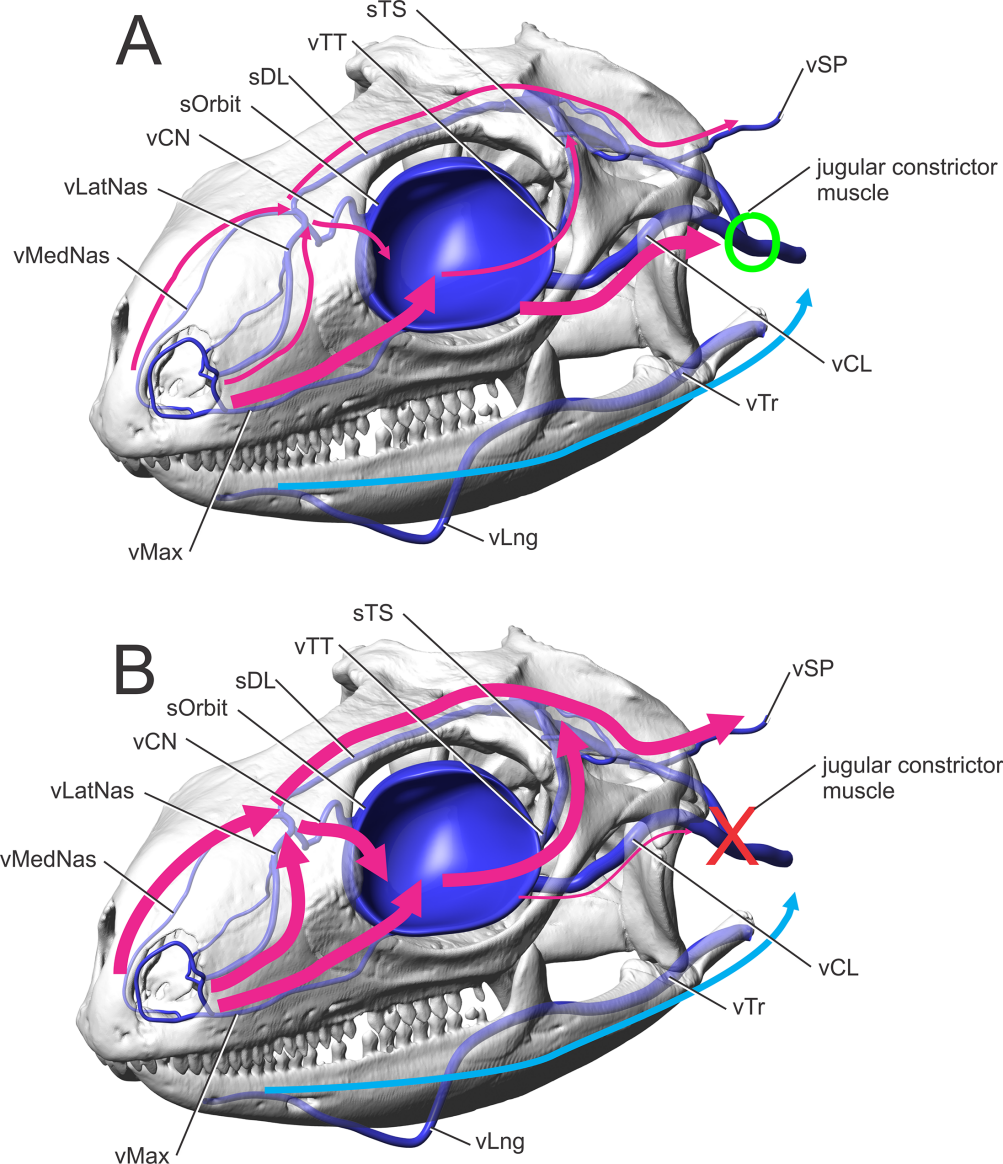
*Iguana iguana* in left rostrodorsolateral oblique view showing diagrammatic veins. (A) Indicates flow pathways with the jugular constrictor muscle (green circle) relaxed and allowing blood to flow into the jugular vein. The thicker lines indicate higher volumes of blood flow through direct pathways to the jugular vein. Thinner lines indicate pathways with less volume of blood flow. (B) Indicates flow pathways with the jugular constrictor muscle (red X) activated, restricting blood to flow into the jugular vein. The thicker lines indicate higher volumes of blood flow through direct pathways to the brain and eyes. Thinner lines indicate pathways with less volume of blood flow.

In iguanas, the nasal cavity and palate are best positioned, at least anatomically, to supply neurosensory tissues with blood that has been thermally adjusted, promoting warming or cooling. The veins rostral to the olfactory bulbs that drain the nasal cavity would allow blood to flow through the dorsal longitudinal sinus or into the orbital sinus, thus regulating temperature of both the brain and the eyeball (S1 File: Views of Figs 10, 11 and 13). Evaporative cooling has been demonstrated in the nasal region, providing both the means of cooling and the ability to deliver cooled blood to the central nervous system. In both birds and crocodilians, as well as squamates, the veins of the nasal cavity are tributaries of the dural sinus system, potentially establishing a plesiomorphic diapsid condition that allows the temperature regulation of neurosensory tissues.

In iguanas, the orbital region is highly vascularized, with the orbital sinus and various arteries passing through this region. The orbital sinus was found to envelop the medial aspect of the orbit, and expanded laterally around the eyeball (S1 File: Views of Figs 10, 11 and 13). Because the nasal region and oral region drain into the orbital sinus, the potential to adjust the temperature of the eyeball is present. By regulating eye temperature, performance of the retina and associated neural tissues may be optimized [59].

The role of the venous drainage of the oral region of iguanas is enigmatic. The venous drainage of the ventral oral region (e.g., tongue) is to the tracheal vein (S1 File: Views of Figs 9, 10 and 11), bypassing the head and draining into the heart, although a small anastomosis with the lateral head vein, via the buccomandibular vein [1], does occur at the level of the jugular constrictor muscle. By bypassing neurosensory tissues of the head, the utility of the oral region in iguanas may be restricted to serving the body. The dorsal oral region (i.e., the palate), however, drains into the orbital sinus [1] and may still influence central nervous system temperature, although the iguana species in this study lacked the dense palatal plexuses of extant archosaurs [50, 60] that would seem to be better suited anatomically for heat exchange.

Additionally, anastomotic connections between the transversotrigeminal vein and the lateral head vein might indicate that cooled blood may also flow into the transverse sinus and thus cool brain tissue (S1 File: Views of Figs 10, 11 and 13). When the internal jugular constrictor muscle contracts, the preferred venous drainage pathways are restricted (Fig 13), pressure increases causing the orbital sinus to expand, and flow is thought to pass through the external jugular vein [4]. Heath [4] called the vertebral veins “small,” but they were found to be relatively large in iguanas. When the jugular constrictor muscle contracts, venous blood flow may pass dorsally through the transversotrigeminal vein, into the transverse sinus and dorsal longitudinal sinus, then into the occipital sinus, and finally into the spinal veins. The caudal cephalic veins are also occluded by the internal jugular constrictor muscle when it contracts [1], blocking drainage into the internal jugular vein and ensuring flow through the spinal veins. This blood then travels through the foramen magnum, though the spinal veins around the spinal cord, essentially cooling (or warming) the entire central nervous system (Fig 13). This hypothesis would indicate a preferential flow pattern that is shunting blood over the central nervous system when the internal jugular constrictor muscle contracts [4, 26]. The jugular constrictor muscle has been shown to contract during both heating [4, 24] and cooling [26], indicating that this muscle may have a role in redirecting venous blood over the central nervous system during thermoregulation.

Birds are thought to control the temperature of neurosensory tissues by using a rete mirabile [29, 45], drawing on cooled venous blood to adjust the temperature of the arterial blood entering the brain and eyes rather than directly influencing the temperature of neurosensory tissue. Lizards and snakes, however, are able to establish a brain-to-body temperature differential without the use of any known rete mirabile. Thus, in the absence of retia, investigations in squamates have focused on the ability of veins to deliver cooled or warmed blood around the head to more *directly* influence head temperature. Ectothermic reptiles are able to establish and maintain a temperature differential of the same magnitude as endothermic birds by directing cooled blood to specific areas of the head. The thermal use of a venous system without retia mirabilia to influence brain temperature in lizards strengthens the hypothesis [60] that in birds veins may play the same direct role in influencing central nervous system temperature and that the avian retia may instead be primarily a physiological device to control the temperature of the eye. Because many diapsids are able to establish and maintain temperature gradients, possibly to assist brain cooling or warming, the use of blood vessels to control the temperature of neurosensory tissues is likely a plesiomorphic condition. This finding would indicate that large-bodied dinosaurs would have employed similar venous flow patterns to adjust temperatures of neurosensory tissues. Because of the large size of many dinosaurs, these mechanisms would be even more critical to the homeostasis of neurosensory tissues.

The vascular patterns in this sample were surprisingly conserved when comparing individuals in the sample to each other and to reports in the literature. There were subtle variations in branching location and pathways, but all of the major branches were predictable and consistent, especially in regards to their osteological correlates. All of the major blood vessels to the nasal, oral, and orbital regions were found to be branches from the respective blood vessel supplying each region. For example, the supraorbital, infraorbital, and ophthalmotemporal arteries all branch from the temporoorbital artery (S1 File: View of Fig 3), the nasal arteries are branches from the supraorbital arteries and rostral cerebral arteries (S1 File: View of Fig 4), and the palate is supplied by arteries from the maxillary and sphenopalatine arteries (S1 File: View of Fig 7). Anastomotic connections between blood vessels were also found to be consistent and predictable. For example, the internal ophthalmic artery between the rostral cerebral and ophthalmotemporal arteries and the anastomosis between the maxillary and sphenopalatine arteries (S1 File: View of Fig 6) were easily recognized. This anatomical consistency is likely in part a reflection of the sample size, as vascular variation is expected and has been found in other vascular studies of other diapsids.

Although the general anatomical connections of the arteries and veins of squamates are known, this study of iguanas has demonstrated the vascular pathways that may be important in delivering blood to neurosensory tissues. Physiological data indicates that sites of thermal exchange are effectively used as a way to influence head or neurosensory tissue temperatures, allowing finer control over behavioral thermoregulation. The anatomical patterns indicate the ability to closely regulate the temperature of the brain and eyes. The veins draining sites of thermal exchange have clear connections with neurosensory tissues. Broad drainage patterns indicate that the jugular constrictor muscle could be acting to redirect blood away from the jugular vein such that, when this muscle contracts, blood from all three sites of thermal exchange can be directed over the central nervous system. This mechanism has to have an impact on the temperature of neurosensory tissues in both warming and cooling, essentially supporting both Heath [4] and Crawford [26]. Activation of the jugular constrictor muscle during basking [1, 5, 24] has the clear potential to warm the central nervous system. Activation of the jugular constrictor muscle during cooling [17] has the clear potential to cool the central nervous system. This fine thermal control of specific tissues in the heads of squamates is probably not feasible with behavioral thermoregulation alone. Squamates thus demonstrate the ability of the cardiovascular system of ectotherms to function as a well-tuned system for delivering thermal energy around the body.

## Supporting Information

S1 File The PDF file S1_File.pdf contains a 3-dimensional model of the skull and blood vessels of an iguana. To begin, click on the iguana skull to active the 3D model. The model in the 3D PDF can be freely rotated and zoomed, structures can be made visible, invisible, or transparent, and the vessels can be identified simply by clicking on them and reading the bolded name at left in the Model Tree. In the center of the Model Tree, a list of views represents the figures found in the text. Clicking on these views will orient the model in the same position as the figures.
(PDF)

## Abbreviations

Anas: anastomosis
aAur: auricular artery
aBas: basilar artery
aCerC: cerebral carotid artery
can: common nasal artery
aGG: genioglossus artery
aHyoM: hyomandibular artery
aIC: internal carotid artery
aIO: infraorbital artery
aLatNas: lateral nasal artery
aMan: mandibular artery
aMax: maxillary artery
aMedNas: medial nasal artery
aMidCer: middle cerebral artery
anasLatNasMax: anastomosis between lateral nasal and maxillary vessels
anasMaxVNas: anastomosis between maxillary and ventral nasal vessels
anasSNVNas: anastomosis between subnarial and ventral nasal vessels
aMan: mandibular artery
aOC: occipital artery
aOpt: ophthalmotemporal artery
aPal: palatine artery
aSN: subnarial artery
aSL: sublingual artery
aSp: sphenopalatine artery
aST: stapedial artery
aSubM: submandibular artery
aTO: temporoorbital artery
aVNas: ventral nasal artery
mPSTs: m. pseudotemporalis superficialis
mAMEP: m. adductor mandibulae externus profundus
mAMES: m. adductor mandibular externus superficialis
sDL: dorsal longitudinal sinus
sOf: olfactory sinus
sOC: occipital sinus
sOrbit: orbital sinus
sPar: parietal sinus
sTr: transverse sinus
vCC: caudal head vein
vCD: dorsal head vein
vCL: lateral head vein
vCN: common nasal vein
vCT: cerebrotectal vein
vOC: occipital vein
vLng: lingual vein
vMedNas: medial nasal vein
vSN: subnarial vein
vTT: transversotrigeminal vein
sTS: transverse sinus
vJug: jugular vein
vLatNas: lateral nasal vein
vMan: mandibular vein
vMax: maxillary vein
vMedNas: medial nasal vein
vNas: nasal vein
vPal: palatine vein
vSpn: spinal vein
vTr: tracheal vein
