# Supplementary figures and images for: Vascular Patterns in Iguanas and Other Squamates: Blood Vessels and Sites of Thermal Exchange
Source: PLoS One. 2015 Oct 14;10(10):e0139215. doi: 10.1371/journal.pone.0139215 (PMC4605690; doi:10.1371/journal.pone.0139215)

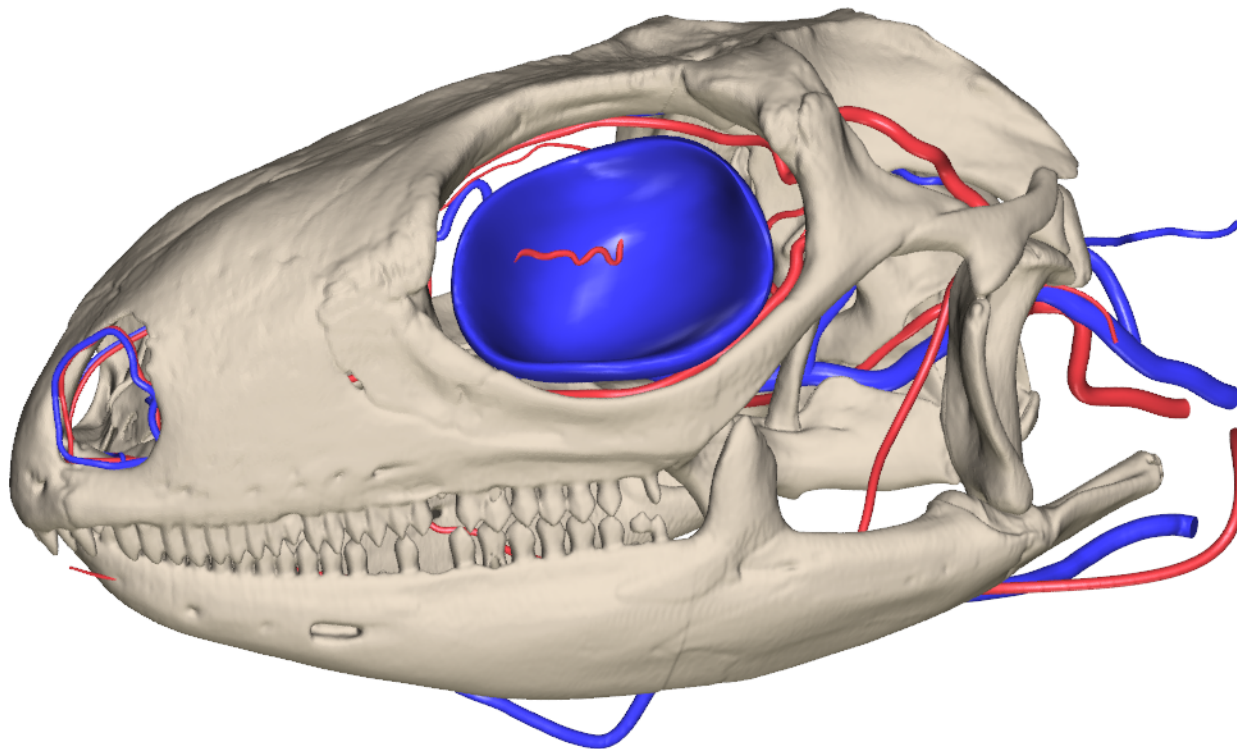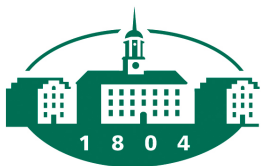

OHIO  
UNIVERSITY

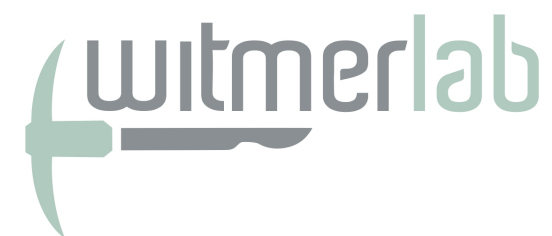

Supplement: S1 File — To begin, click on the iguana skull to active the 3D model. The model in the 3D PDF can be freely rotated and zoomed, structures can be made visible, invisible, or transparent, and the vessels can be identified simply by clicking on them and reading the bolded name at left in the Model Tree. In the center of the Model Tree, a list of views represents the figures found in the text. Clicking on these views will orient the model in the same position as the figures. (PDF) [file pone.0139215.s001.pdf]
